# Valorization of coal fly ash into a magnetic Fe₃O₄-decorated composite for Cu(II) removal from aqueous systems

**DOI:** 10.1038/s41598-026-41916-2

**Published:** 2026-03-05

**Authors:** Sulistyo Saputro, Lina Mahardiani, Mohammad Masykuri, Sri Yamtinah, Amun Amri, Cindy Enrica Novitasari, Tiwi Srilingi, Cinthia Cinthia

**Affiliations:** 1 Universitas Sebelas Maret, Surakarta, Indonesia; 2 University of Riau, Pekanbaru, Indonesia

**Keywords:** Coal fly ash, Fe₃O₄-decorated fly ash, Magnetic adsorbent, Cu(II) removal, Waste valorization, Chemistry, Environmental sciences, Materials science

## Abstract

Coal fly ash is generated in large quantities by coal-fired power plants and is commonly disposed of as waste despite containing reactive mineral phases. This study investigates the valorization of coal fly ash into a magnetic Fe₃O₄-decorated composite for Cu(II) removal from aqueous systems, linking waste reutilization with the development of low-cost and magnetically recoverable materials for water treatment. The composite was synthesized via a coprecipitation–thermal treatment route, promoting the deposition of iron oxide on chemically activated fly ash. The physicochemical properties of the resulting material were characterized using X-ray fluorescence (XRF), X-ray diffraction (XRD), Fourier transform infrared spectroscopy (FTIR), scanning electron microscopy (SEM), transmission electron microscopy (TEM), and N₂ adsorption–desorption (BET) analysis. Cu(II) concentrations before and after adsorption were quantified by atomic absorption spectroscopy (AAS). Batch adsorption experiments were performed under fixed experimental conditions to evaluate the influence of composite composition and contact time on Cu(II) removal. The Fe₃O₄-decorated fly ash composite exhibited enhanced magnetic recoverability and improved Cu(II) removal compared with non-magnetic fly ash, enabling efficient solid–liquid separation. Under the selected experimental conditions, approximately 80% Cu(II) removal was achieved, yielding an adsorption capacity of ~0.32 mg g⁻^1^ at an initial Cu(II) concentration of 4 mg L⁻^1^ and a contact time of 120 min. Kinetic analysis indicated that Cu(II) uptake followed pseudo-second-order behavior, suggesting chemisorption-controlled adsorption. The developed material is positioned as a low-cost, sustainable, and magnetically recoverable adsorbent for coal fly ash valorization rather than as a high-capacity adsorbent. The findings highlight the role of chemical activation and magnetic modification in tailoring fly ash-based composites and provide a basis for further performance optimization.

**Supplementary Information:**

The online version contains supplementary material available at 10.1038/s41598-026-41916-2.

## Introduction

Coal fly ash (CFA) is a solid residue generated from coal combustion in the form of very fine particles and is typically dark gray to black in color. The management of coal fly ash remains a critical environmental issue, particularly in countries where coal-fired power plants continue to serve as major energy sources. Coal combustion has been identified as one of the most significant anthropogenic contributors to local and regional air pollution, with documented adverse impacts on ecosystems and human health^1^. The steam power plant sector represents the dominant source of coal fly ash generation, as coal is extensively used as its primary fuel^2^. Fly ash particles released from power plants can be transported by wind and dispersed into surrounding environments, where inhalation exposure has been associated with respiratory disorders among nearby populations^3^. Communities residing in the vicinity of coal-fired power plants have been reported to experience increased risks of respiratory hospitalization, long-term lung function decline, and premature mortality linked to particulate exposure^4^. In Indonesia, coal production reached approximately 836 million tonnes in 2024, generating substantial quantities of fly ash annually^5^. The volume of fly ash produced greatly exceeds its current utilization capacity, leading to widespread disposal through landfilling or uncontrolled stockpiling, which further intensifies associated environmental risks.

Extensive research efforts have explored the potential utilization of coal fly ash in various engineering and environmental applications to mitigate its environmental burden and enhance resource efficiency^6–10^. Reported applications include its incorporation as a supplementary cementitious material in the cement industry, use in road construction, brick manufacturing, land reclamation, and landfill cover systems. Coal fly ash has also attracted increasing attention as a precursor material for adsorption processes, rubber filler formulations, and as a raw material for the synthesis of mesoporous silica and zeolitic materials^11–15^. These valorization strategies aim not only to reduce the environmental footprint associated with fly ash disposal but also to exploit its aluminosilicate-rich composition for functional material development.

Another important application of coal fly ash is its use as a low-cost adsorbent for the removal of heavy metals, organic contaminants, and dyes from wastewater, taking advantage of its abundance, low cost, and potential environmental benefits^16^^,^^17^. Adsorbents with mesoporous structures and well-developed pore networks generally exhibit superior performance in water purification processes^18^. Raw coal fly ash typically possesses a relatively low specific surface area and an unfavorable pore structure, which limits its intrinsic adsorption capacity. To overcome these limitations, various chemical and physical modification strategies have been explored to enhance the adsorption efficiency of coal fly ash toward metal ions. Such strategies include acid or alkaline activation, surface functionalization, and composite formation with metal oxides^19^^,^^16^. Previous studies have reported that modification of fly ash with metal oxides and polymeric additives, such as polyethylene glycol (PEG), can improve adsorption performance by promoting pore formation and regulating pore size distribution, thereby increasing the accessibility of active adsorption sites^20^^,^^21^.

Coal fly ash has therefore attracted increasing attention as a precursor material for adsorbents in water treatment applications. Its aluminosilicate-rich composition provides a chemically stable framework that can be further modified through activation or surface decoration to enhance affinity toward dissolved metal ions. Incorporation of magnetic iron oxide phases into fly ash-based adsorbents has been widely investigated as a means to impart magnetic separability, facilitating solid–liquid separation and improving operational practicality in adsorption-based treatment systems. In such materials, iron oxide is commonly present on the fly ash surface as discrete domains or surface-decorated phases rather than as a continuous and uniform shell layer. These materials are more appropriately described as magnetic fly ash composites or surface-decorated structures rather than idealized core–shell architectures, particularly in the absence of direct transmission electron microscopy (TEM) evidence for concentric shell formation.

Magnetite (Fe₃O₄), one of the most widely studied magnetic materials, has attracted considerable attention for applications in magnetic resonance imaging, sensors, and adsorption-based separation processes^22^. Magnetite can be synthesized via coprecipitation methods that offer high yield, relatively simple processing, and favorable atomic economy^23^. Its intrinsic magnetic properties enable rapid separation and recovery under an external magnetic field, which is particularly advantageous for adsorption applications^24^. Fe₃O₄ nanoparticles also exhibit a relatively high surface area and abundant surface-active sites, yet their strong tendency to aggregate during synthesis and application can significantly reduce the number of accessible adsorption sites and limit adsorption performance. To mitigate this limitation, Fe₃O₄ nanoparticles are commonly combined with organic or inorganic support materials to suppress aggregation and improve structural stability^25^. In such composite systems, Fe₃O₄ is frequently present as surface-decorated domains or core–shell-like configurations, which preserve magnetic separation performance while reducing direct particle–particle adhesion and uncontrolled aggregation of magnetite nanoparticles^26^.

The concept of core–shell or surface-decorated architectures has been widely explored as an effective structural design in functional materials. Such configurations can increase accessible surface area, generate additional active sites, and enhance performance in applications including catalysis, drug delivery, and adsorption^24^^,^^27^^,^^28^. In adsorption systems, the presence of a supporting matrix can improve dispersion of the active phase and provide complementary surface functionalities, while magnetic cores enable facile separation and reuse^29^. In practical mineral-based composites, ideal concentric core–shell structures are not always achieved, and iron oxide phases are frequently distributed as surface-decorated domains rather than as uniform shells. Accurate identification of such structural configurations requires nanoscale characterization, particularly transmission electron microscopy (TEM), which is not consistently reported in fly ash–based magnetic adsorbents^30^.

Recent studies have highlighted that adsorption performance toward heavy metal ions is strongly governed by material microstructure, surface functional groups, and surface chemistry^31^^,^^32^. Bio-derived and mineral-based adsorbents modified through chemical activation or composite formation have demonstrated promising performance for Cu(II) and other metal ion removal, particularly when surface hydroxyl groups and metal–oxygen functionalities are abundant^33^^,^^34^. These findings underscore the importance of developing low-cost, structurally characterized, and magnetically recoverable adsorbents with tailored surface properties. In this context, Fe₃O₄-decorated fly ash composites represent a promising class of materials for wastewater treatment applications, combining the adsorption potential of activated fly ash with the magnetic separability of iron oxide phases.

Cu(II) ions are considered among the most hazardous heavy metal contaminants due to their redox activity and high mobility, and excessive exposure has been associated with various adverse health effects, including organ damage and carcinogenic risks^35^. Reducing Cu(II) concentrations in wastewater, particularly in industrial effluents, is therefore essential for mitigating its environmental and health impacts. Numerous treatment technologies have been developed for Cu(II)-containing wastewater, such as membrane filtration, ion exchange, chemical precipitation, coagulation–flocculation, and flotation^36^. Among these approaches, adsorption remains attractive due to its operational simplicity, cost effectiveness, and flexibility^37^.

In this study, coal fly ash was valorized into a magnetic Fe₃O₄-decorated composite via a coprecipitation–thermal treatment route to develop a low-cost and magnetically recoverable material for Cu(II) removal from aqueous systems. The investigation examines the influence of chemical activation using NaOH and HCl, Fe₃O₄ deposition, and composite composition on the structural features, surface chemistry, and adsorption behavior of the resulting material. Material characterization was performed using X-ray fluorescence (XRF) to determine bulk chemical composition, X-ray diffraction (XRD) for phase identification, Fourier transform infrared spectroscopy (FTIR) for functional group analysis, scanning electron microscopy (SEM) and transmission electron microscopy (TEM) for morphological evaluation, and N₂ adsorption–desorption (BET) analysis to assess textural properties. Batch adsorption experiments, coupled with atomic absorption spectroscopy (AAS), were conducted to assess Cu(II) removal efficiency and adsorption kinetics under controlled laboratory conditions. The study highlights the potential of fly ash valorization through magnetic modification and emphasizes structure–performance relationships, while explicitly recognizing limitations associated with single-concentration adsorption conditions and the absence of comprehensive equilibrium isotherm evaluation.

## Materials and methods

### Chemicals

Coal fly ash used in this study was collected from a coal-fired steam power plant (PLTU) located in Adipala, Cilacap, Indonesia. The fly ash was obtained directly from the electrostatic precipitator unit of the plant, followed by drying and washing steps prior to use. Coal fly ash is known to contain a variety of inorganic constituents, including major oxides such as Si, Al, Fe, Ca, and Mg, along with minor elements depending on coal source and combustion conditions. In the present work, the bulk elemental and oxide composition of the raw fly ash was determined using X-ray fluorescence (XRF) analysis, as described in the Characterization Techniques section. The XRF results were used to assess the overall chemical composition and to evaluate the presence of major and minor elements relevant to adsorption performance and environmental considerations. Detailed trace-level analysis using techniques such as ICP-OES or ICP-MS was not conducted. The mobility or leaching behavior of trace elements under aqueous conditions was not evaluated and is acknowledged as beyond the scope of this study. Ferric nitrate nonahydrate (Fe(NO₃)₃·9H₂O), ferrous chloride pentahydrate (FeCl₂·5H₂O), sodium hydroxide (NaOH), hydrochloric acid (HCl), and polyethylene glycol (PEG 4000) were purchased from PT Merck Indonesia. All chemicals were of analytical grade and were used as received without further purification.

### Characterization techniques

#### BET analysis

N₂ adsorption–desorption analysis was conducted to evaluate the textural properties of the FeOCZ sample (Fe₃O₄-decorated fly ash composite) using a NOVA 800 Physisorption Analyzer (Anton Paar). Prior to analysis, the sample was vacuum-degassed at 200 °C for 6 h to remove physically adsorbed moisture and impurities. Nitrogen adsorption–desorption isotherms were recorded at 77.35 K. The specific surface area was determined using the multipoint Brunauer–Emmett–Teller (BET) method within the linear relative pressure range of p/p₀ ≈ 0.01–0.30. The total pore volume was estimated from the amount of nitrogen adsorbed at a relative pressure of p/p₀ ≈ 0.986, while the pore size distribution and mesopore characteristics were calculated using the Barrett–Joyner–Halenda (BJH) method.

#### FTIR spectroscopy

Fourier transform infrared (FTIR) spectra were recorded using a Shimadzu IRSpirit-T FTIR spectrometer (Shimadzu Corporation, Kyoto, Japan). Measurements were conducted over the wavenumber range of 4000–400 cm⁻^1^ using the KBr pellet method. The spectra were collected with a resolution of 4 cm⁻^1^ and 32 scans per sample.

#### XRD analysis

X-ray diffraction (XRD) analysis was carried out using a PANalytical Empyrean X-ray diffractometer (Malvern PANalytical B.V., Almelo, The Netherlands) equipped with Cu Kα radiation (λ = 1.5406 Å). Diffraction patterns were collected over a 2θ range of 10–80° at an operating voltage of 40 kV and a current of 30 mA.

#### XRF analysis

X-ray fluorescence (XRF) analysis was employed to determine the bulk elemental composition of the fly ash samples. Prior to analysis, the fly ash was washed with deionized water to remove water-soluble impurities, followed by filtration and oven drying at 110 °C for 3 h to eliminate residual moisture. The dried material was then sieved through a 100-mesh sieve to obtain a uniform particle size suitable for compositional analysis. XRF measurements were subsequently carried out using a calibrated XRF spectrometer to identify the major oxide components and elemental constituents of the fly ash.

#### SEM analysis

Scanning electron microscopy (SEM) analysis was carried out using a JEOL JSM-IT300 scanning electron microscope (JEOL Ltd., Tokyo, Japan) operated at an accelerating voltage of 10–20 kV. Prior to observation, the samples were sputter-coated with a thin gold layer to improve surface conductivity and minimize charging effects. SEM images were acquired at various magnifications to examine particle morphology and surface texture.

#### TEM analysis

Transmission electron microscopy (TEM) was employed to examine the nanoscale morphology and internal structure of the FeOCZ composite. TEM observations were performed using a transmission electron microscope operated at an appropriate accelerating voltage. The samples were ultrasonically dispersed in ethanol, and a drop of the suspension was deposited onto a carbon-coated copper grid and dried prior to analysis. TEM images were acquired at different magnifications to evaluate the spatial distribution of Fe₃O₄ domains on the fly ash matrix and to assess the presence of surface-decorated or core–shell-like structural features.

### Atomic Absorption Spectroscopy (AAS) analysis

Atomic absorption spectroscopy (AAS) was used to determine Cu(II) concentrations in aqueous solutions before and after adsorption experiments. Measurements were carried out using an AAS instrument operated under appropriate analytical conditions for Cu detection. Calibration curves were constructed using standard Cu(II) solutions over a concentration range of 5–30 mg L⁻^1^. The absorbance values obtained for the samples were converted to Cu(II) concentrations using the calibration equation. All measurements were performed in replicate to ensure data reliability.

### Fly ash preparation and activation

Coal fly ash was used as the starting material and subjected to chemical activation using NaOH and HCl to modify its surface properties prior to composite formation. Fly ash was first sieved through a 100-mesh sieve to remove coarse particles and visible impurities, ensuring a more uniform particle size suitable for activation. The sieved fraction was washed several times with distilled water and dried in a desiccator to obtain dry fly ash powder. For the activation process, 100 g of dried fly ash was mixed with 100 mL of either 1.5 M NaOH or 1.5 M HCl in a round-bottom flask and refluxed at 90 °C for 6 h under continuous stirring. After chemical treatment, the solid phase was separated by filtration and washed repeatedly with distilled water until the filtrate reached approximately neutral pH (pH ≈ 7). The neutralized fly ash samples were dried in a desiccator and then heated at 120 °C for 2 h to obtain the chemically activated fly ash. NaOH-activated fly ash and HCl-activated fly ash prepared under these conditions were used as supports for Fe₃O₄ deposition in the subsequent composite synthesis.

### Preparation of magnetic Fe₃O₄-decorated fly ash composite

As a preliminary step for composite preparation, Fe₃O₄ nanoparticles were synthesized by a coprecipitation method using Fe(NO₃)₃·9H₂O and FeCl₂·5H₂O as iron precursors with a molar ratio of Fe^3^⁺:Fe^2^⁺ = 1:2. This precursor ratio differs from the conventional 2:1 ratio commonly used for bulk magnetite synthesis and was selected to accommodate the presence of iron-bearing phases naturally contained in the fly ash matrix. The iron salts were dissolved in distilled water and stirred at 400 rpm for 3 min using a magnetic stirrer. A 2.5 M NaOH solution was then added dropwise until the formation of a black precipitate was observed and the pH reached approximately 12. The resulting precipitate was separated using an external magnet, washed three times with distilled water to remove residual ions, and dried in an oven at 80 °C for 4 h. The obtained Fe₃O₄ nanoparticles were subsequently combined with fly ash to produce Fe₃O₄-decorated fly ash composites, as described in the following preparation steps.

### Preparation of Fe₃O₄-decorated fly ash composite

The Fe₃O₄-decorated fly ash composite was prepared by combining PEG 4000, Fe₃O₄, and chemically activated fly ash. PEG 4000 was first heated on a hotplate at 60 °C under magnetic stirring for 10 min until a homogeneous molten phase was obtained. PEG 4000 was used as a processing aid to facilitate dispersion of the magnetic phase during composite formation. Fe₃O₄ powder was then added to the molten PEG 4000 and mixed thoroughly to promote uniform distribution of the magnetic particles. The chemically activated fly ash was introduced into the mixture and stirred to promote contact between Fe₃O₄ and the fly ash particle surfaces. The resulting mixture was obtained in the form of a solid precipitate. The precipitate was transferred to a furnace and thermally treated at 400 °C for 3 h to obtain the Fe₃O₄-decorated fly ash composite. The same procedure was applied using different fly ash mass variations to examine the effect of composite composition on material properties.

### Performance test of Fe₃O₄-decorated fly ash adsorbent using AAS

The adsorption performance of Fe₃O₄-decorated fly ash composites was evaluated using atomic absorption spectroscopy (AAS) to quantify Cu(II) removal under controlled batch conditions. Several adsorbent formulations were prepared by varying the mass ratios of fly ash and polyethylene glycol (PEG 4000), while the Fe₃O₄ content was kept constant. PEG 4000 was used consistently as a dispersing and pore-modifying agent during composite preparation. Batch adsorption experiments were conducted by adding 0.1 g of adsorbent to 100 mL of Cu(II) aqueous solution with a fixed initial concentration of 4 mg L⁻^1^. The suspensions were agitated at room temperature (25 ± 2 °C) using a mechanical shaker at a constant speed of 150 rpm. The effect of contact time on Cu(II) removal was evaluated by withdrawing samples at predetermined time intervals of 0, 15, 30, 45, 60, 90, and 120 min. After each contact time, the suspension was filtered to separate the solid adsorbent from the liquid phase. The residual Cu(II) concentration in the filtrate was determined by atomic absorption spectroscopy (AAS). The measured concentrations were used to calculate Cu(II) removal efficiency and adsorption capacity as a function of contact time. All adsorption performance tests were carried out under identical experimental conditions to allow direct comparison among the different adsorbent formulations. The adsorption experiments were limited to a single initial Cu(II) concentration and were intended to evaluate adsorption kinetics and comparative removal performance rather than to establish equilibrium adsorption isotherms based on multiple concentration levels.

### Adsorption experiments

Adsorption experiments were conducted in batch mode to evaluate the kinetic and equilibrium behavior Adsorption experiments were conducted in batch mode to evaluate the adsorption performance and kinetic behavior of Cu(II) uptake by Fe₃O₄-decorated fly ash composites. In each experiment, a known mass of adsorbent (0.1 g) was added to 100 mL of Cu(II) aqueous solution in conical flasks and agitated using a mechanical shaker at a constant shaking speed of 150 rpm. The solution pH was adjusted to approximately 7 using dilute NaOH or HCl solutions, and all experiments were carried out at room temperature (25 ± 2 °C). For kinetic studies, samples were withdrawn at predetermined time intervals, and the residual Cu(II) concentration was analyzed to determine adsorption capacity as a function of contact time. Adsorption experiments were conducted under fixed initial Cu(II) concentration conditions to enable comparative evaluation of adsorption performance and kinetic behavior among the investigated samples. Equilibrium adsorption capacity was determined by allowing a contact time of 120 min, which was sufficient to reach adsorption equilibrium under the selected experimental conditions.

### Adsorption calculations and kinetic models

#### Adsorption calculations

The adsorption performance of the Fe₃O₄@fly ash composites was evaluated by determining the adsorption capacity at different contact times and at equilibrium. The amount of Cu(II) adsorbed per unit mass of adsorbent was calculated using the following standard equations, which are widely applied in adsorption studies^38^^,^^39^.

Adsorption Capacity at Time *t* (${q}_{t}$)

$${q}_{t} = \frac{\left({C}_{0}-{C}_{t}\right)\times V}{m}$$

Adsorption Capacity at Equilibrium (${q}_{e}$)

$${q}_{e} = \frac{\left({C}_{0}-{C}_{e}\right)\times V}{m}$$

Where:

${q}_{t}$ = adsorption capacity at time t (mg g⁻^1^)

${q}_{e}$= adsorption capacity at equilibrium (mg g⁻^1^)

${C}_{0}$= initial adsorbate concentration (mg L⁻^1^)

${C}_{t}$= adsorbate concentration at time t (mg L⁻^1^)

${C}_{e}$= equilibrium adsorbate concentration (mg L⁻^1^)

$V$ = volume of the solution (L)

$m$ = mass of dry adsorbent (g)

#### Adsorption kinetic models

The adsorption kinetics were evaluated using pseudo-first-order (PFO) and pseudo-second-order (PSO) kinetic models to describe the rate and mechanism of Cu(II) uptake by the Fe₃O₄@fly ash composites.

Pseudo–First–Order (PFO) Model

$${q}_{t}= {q}_{e} \left(1- {e}^{{-k}_{1}t}\right)$$

Where:

${k}_{1}$ is PFO rate constant (min⁻^1^)

Pseudo–Second–Order (PSO) Model

$${q}_{t}= \frac{{q}_{e}^{2}{k}_{2}t}{1+ {q}_{e}{k}_{2}t}$$

Where:

${k}_{2}$ is the PSO rate constant (g mg⁻^1^ min⁻^1^)

The kinetic parameters were obtained by regression analysis of the experimental data. Comparison between kinetic models was based on the calculated parameters and correlation coefficients.

#### Intraparticle diffusion (IPD) model

To qualitatively examine the contribution of diffusion-controlled processes during adsorption, the intraparticle diffusion (IPD) model proposed by Weber and Morris was applied:

$${q}_{t}= {k}_{id}{t}^{\raisebox{1ex}{$1$}\!\left/ \!\raisebox{-1ex}{$2$}\right.}+C$$

with parameters:

${k}_{id}$= intraparticle diffusion rate constant (mg g⁻^1^ min⁻^10.^^5^)

$C$ = boundary layer thickness

The IPD model was used as a mechanistic reference to illustrate possible diffusion contributions during adsorption. Quantitative multi-linear fitting and stage-wise diffusion analysis were not performed due to insufficient kinetic data points to support reliable interpretation. Textural properties of the Fe₃O₄@fly ash composite, including specific surface area, pore volume, and pore size distribution, were determined by N₂ adsorption–desorption (BET) analysis. The obtained quantitative textural parameters, together with complementary evidence from SEM, TEM, XRD, XRF, and FTIR analyses, were used to support interpretation of the adsorption behavior.

## Results and discussion

### Textural properties of FeOCZ (BET analysis)

**Fig. 1:**
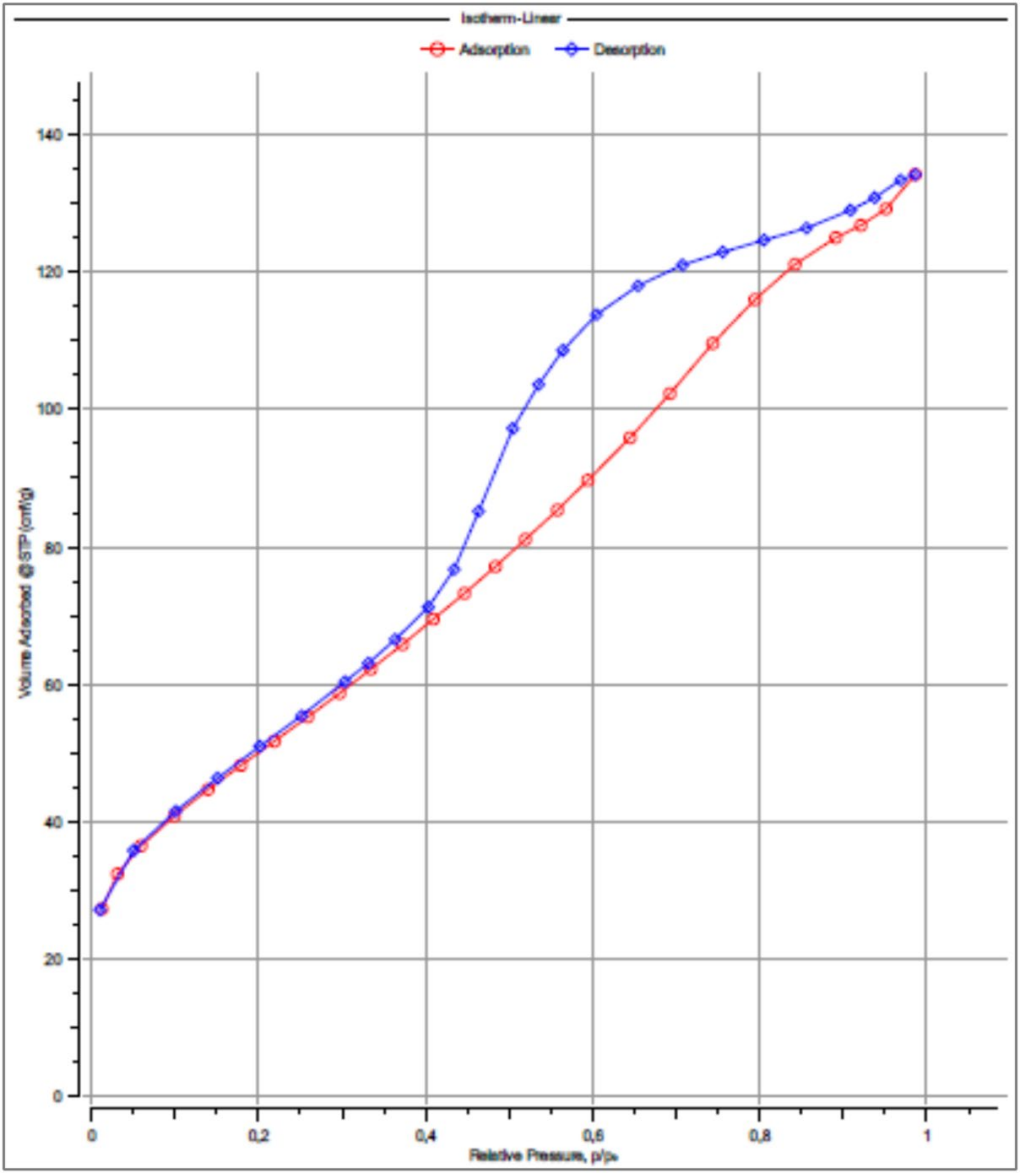
N₂ adsorption–desorption isotherm of FeOCZ measured at 77.35 K, showing a hysteresis loop indicative of mesoporous characteristics.

The N₂ adsorption–desorption behavior of FeOCZ was investigated at 77.35 K, and the resulting isotherm is presented in Figure 1.

As shown in Figure 1, the N₂ adsorption–desorption isotherm of FeOCZ exhibits features characteristic of mesoporous materials. At low relative pressures (p/p₀ < 0.1), nitrogen uptake increases gradually, indicating monolayer adsorption on the accessible surface sites of the composite. With increasing relative pressure in the intermediate region, a more pronounced increase in adsorption is observed, which is associated with multilayer adsorption and the onset of capillary condensation within mesoporous structures. The presence of a clear hysteresis loop between the adsorption and desorption branches reflects differences in the condensation and evaporation pathways of nitrogen within mesopores, providing strong evidence that the pore structure of FeOCZ is dominated by mesoporous characteristics.

**Fig. 2:**
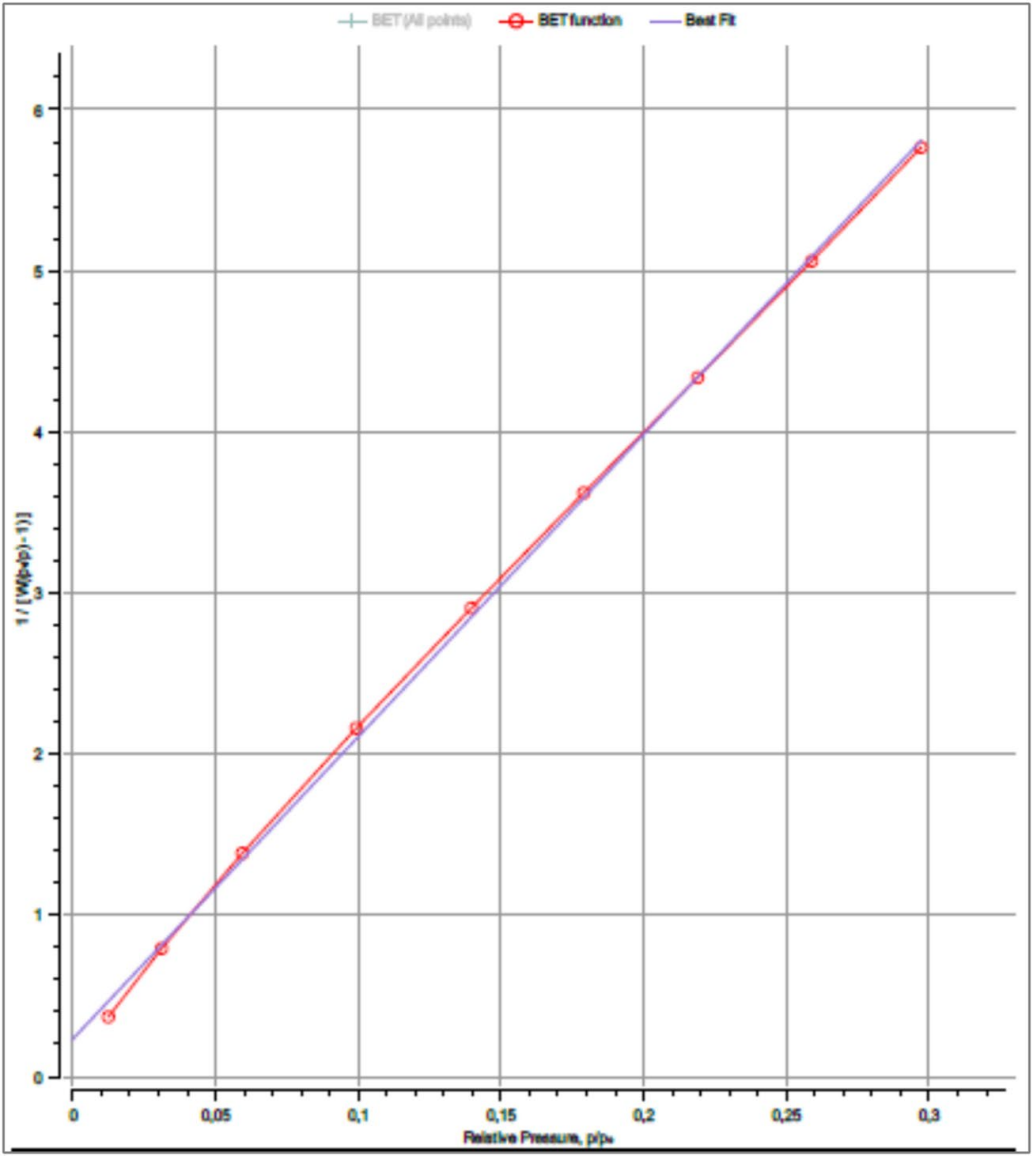
Multipoint BET plot of FeOCZ showing excellent linearity (p/p₀ ≈ 0.01–0.30) used for calculation of the specific surface area.

This qualitative interpretation based on the isotherm shape is further supported by quantitative surface area analysis. The specific surface area of FeOCZ was therefore determined using the multipoint Brunauer–Emmett–Teller (BET) method within the linear relative pressure range of p/p₀ ≈ 0.01–0.30, and the corresponding BET plot is presented in Figure 2.

As shown in Figure 2, the multipoint BET plot of FeOCZ exhibits excellent linearity within the selected relative pressure range of p/p₀ ≈ 0.01–0.30, indicating that the BET model is appropriately applied for surface area determination. The high correlation coefficient (r = 0.9996) reflects a strong linear relationship between the transformed BET function and the relative pressure, confirming the reliability of the experimental adsorption data and the validity of the multipoint BET fitting. Based on this analysis, the BET specific surface area of FeOCZ was calculated to be 183.397 m^2^ g⁻^1^. This relatively high surface area demonstrates that the magnetic modification and composite formation processes effectively increased the accessible surface of the fly ash-based material. Such an increase in surface area is expected to enhance the availability of adsorption sites, thereby supporting improved interaction between the adsorbent surface and adsorbate species during the adsorption process. In addition to surface area, further quantitative textural parameters derived from BET and BJH analyses, including pore volume and pore size characteristics, provide complementary insight into the pore structure of FeOCZ. These parameters are summarized in Table 1, which presents a comprehensive overview of the textural properties obtained from N₂ adsorption–desorption analysis at 77.35 K.

**Table 1:** Textural properties of FeOCZ derived from N₂ adsorption–desorption (BET) analysis at 77.35 K.

**Parameter**	**Value**	**Method**
BET surface area, SBET	183.397 m^2^ g⁻^1^	Multipoint BET
BET slope	18.757	BET plot
BET intercept	0.2325	BET plot
Correlation coefficient (R^2^)	0.9992	BET plot
BET constant (C)	81.65	BET equation
Total pore volume	0.2081 cm^3^ g⁻^1^	p/p₀ ≈ 0.986
Average pore diameter	4.54 nm	Instrument summary
BJH pore diameter (adsorption)	3.74 nm	BJH method
BJH pore volume (adsorption)	0.1382 cm^3^ g⁻^1^	BJH method
BJH pore diameter (desorption)	3.60 nm	BJH method
BJH pore volume (desorption)	0.1790 cm^3^ g⁻^1^	BJH method

**Fig. 3:**
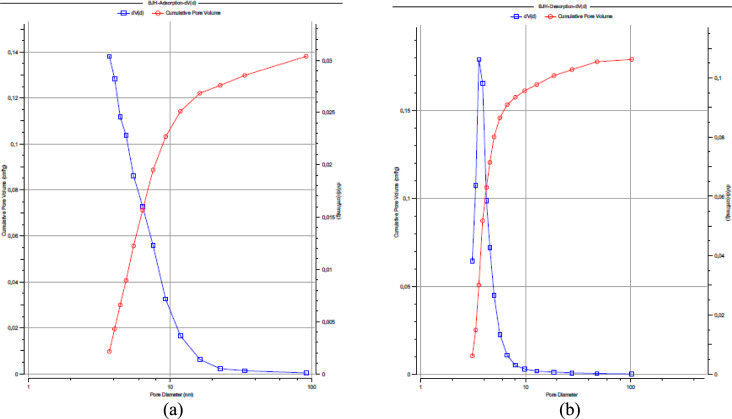
BJH pore size distribution and cumulative pore volume of FeOCZ derived from (**a**) adsorption and (**b**) desorption branches of the N₂ adsorption–desorption isotherm.

As summarized in Table 1, the quantitative textural parameters derived from N₂ adsorption–desorption analysis further substantiate the mesoporous characteristics of FeOCZ. In addition to the high BET specific surface area (183.397 m^2^ g⁻^1^), FeOCZ exhibits a total pore volume of 0.2081 cm^3^ g⁻^1^, indicating the presence of a well-developed pore network capable of accommodating adsorbate molecules. The average pore diameter of 4.54 nm places the dominant pore size distinctly within the mesoporous range, which is consistent with the hysteresis behavior observed in the adsorption–desorption isotherm. The pore size parameters obtained from the BJH method show pore diameters of approximately 3.6–3.7 nm for the adsorption and desorption branches. The close agreement between these values suggests a relatively uniform mesoporous structure, while the slight differences between the two branches can be attributed to variations in pore geometry and the condensation–evaporation mechanisms inherent to mesoporous materials. Such pore characteristics are advantageous for adsorption processes, as they promote efficient mass transfer and reduce diffusion resistance within the pore channels. To provide a more detailed visualization of the pore size distribution, the BJH pore size distribution and cumulative pore volume derived from both adsorption and desorption branches are presented in Figure 3. These distributions further illustrate the dominance of mesopores in FeOCZ and complement the quantitative parameters summarized in Table 1.

As shown in Figure 3, the BJH pore size distribution and cumulative pore volume curves derived from both adsorption and desorption branches provide further insight into the pore structure of FeOCZ. The incremental pore volume distribution (dV/dD) exhibits a pronounced contribution within the mesoporous range, indicating that a significant portion of the pore volume is associated with pores of several nanometers in diameter. This observation is consistent with the BJH pore diameters summarized in Table 1, which fall within the range of approximately 3.6–3.7 nm. The cumulative pore volume profiles in Figure 3 increase rapidly at small-to-intermediate pore diameters and gradually approach a plateau at larger diameters, suggesting that mesopores contribute predominantly to the total pore volume. The similarity between the distributions obtained from the adsorption and desorption branches indicates that the mesoporous network is represented consistently by both branches, while minor deviations can be attributed to hysteresis effects and pore-geometry-dependent condensation and evaporation phenomena typical of mesoporous materials. The BJH results complement the adsorption–desorption isotherm (Figure 1) and the multipoint BET analysis (Figure 2) by providing a detailed visualization of the pore size distribution associated with the accessible surface area of FeOCZ. These textural characteristics establish a structural framework for adsorption by facilitating adsorbate accessibility and mass transport within the pore network. To further characterize the surface chemical functionalities that may influence adsorbate interactions, FTIR spectroscopy was employed to identify functional groups present on the FeOCZ surface.

### FTIR characterization

**Fig. 4:**
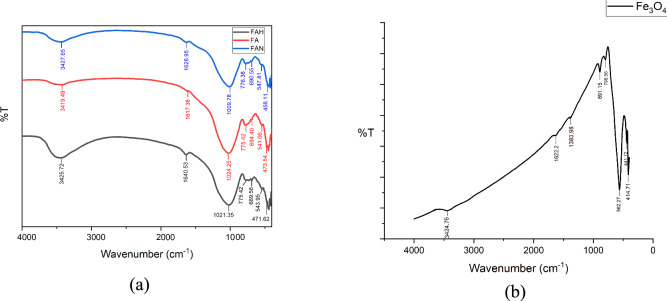
FTIR spectra of: (**a**) fly ash (FA), NaOH activated fly ash (FAN), HCl activated fly ash (FAH); and (**b**) Fe_3_O_4_.

FTIR spectroscopy was employed to examine the surface chemical functionalities of the fly ash–based materials and Fe₃O₄. The resulting spectra shown in Figure 4 provide information on the functional groups present on the material surfaces that are relevant to adsorption behavior.

Figure 4 shows the FTIR spectra of fly ash (FA), NaOH-activated fly ash (FAN), HCl-activated fly ash (FAH), and Fe₃O₄. As shown in Figure 4(a), the spectra of FA, FAN, and FAH display strong and broad absorption bands at 3419.94 cm⁻^1^ for FA, 3425.72 cm⁻^1^ for FAN, and 3427.65 cm⁻^1^ for FAH, which are attributed to the stretching vibration of O–H groups. Together with the absorption bands observed at 1617.38 cm⁻^1^ for FA, 1640.53 cm⁻^1^ for FAN, and 1628.95 cm⁻^1^ for FAH, assigned to H–O–H bending vibrations, these features indicate the presence of hydrogen-bonded water molecules (H₂O) and surface hydroxyl groups on the fly ash surface. The presence of surface O–H groups is particularly important, as they can serve as active sites for Cu(II) complexation or ion-exchange interactions during the adsorption process.

The absorption bands located at 1024.25 cm⁻^1^ for FA, 1021.35 cm⁻^1^ for FAN, and 1009.78 cm⁻^1^ for FAH correspond to asymmetric stretching vibrations of Si–O or Al–O bonds within SiO₄ or AlO₄ tetrahedra, typically observed in the range of 1250–950 cm⁻^1^. These bands confirm that the aluminosilicate framework of fly ash is preserved after chemical activation. The slight shifts in wavenumber observed for FAN and FAH suggest modifications in the local bonding environment, which are commonly associated with partial dissolution or rearrangement of silicate and aluminate phases. Such structural modifications can contribute to an increased number of accessible surface sites, thereby enhancing adsorption potential. In the lower wavenumber region, absorption bands in the range of 800–500 cm⁻^1^ are associated with symmetric stretching vibrations of Si–O–Si and Al–O–Si bonds, while bands below 500 cm⁻^1^ are attributed to bending vibrations of Si–O–Si and O–Si–O linkages^40^^,^^41^. These features further confirm the dominance of silicate and aluminosilicate phases in the fly ash matrix.

The FTIR spectrum of Fe₃O₄ shown in Figure 4(b) exhibits characteristic absorption bands in the low-wavenumber region, which are assigned to Fe–O lattice vibrations and confirm the formation of magnetite. In addition, O–H stretching vibrations at 3424.76 cm⁻^1^, O–H bending at 1622.2 cm⁻^1^, and O–H bending at 1383.98 cm⁻^1^ indicate the presence of surface hydroxyl groups on Fe₃O₄. Bands at 798.56 cm⁻^1^ correspond to octahedrally coordinated Fe^3^⁺–O bonds, while absorption bands at 441.72 cm⁻^1^ and 414.71 cm⁻^1^ are attributed to octahedrally and tetrahedrally coordinated Fe^2^⁺–O bonds, respectively^42–44^. These characteristic bands are consistent with the formation of spinel-type Fe₃O₄, confirming its role as the magnetic phase in the composite system.

**Fig. 5:**
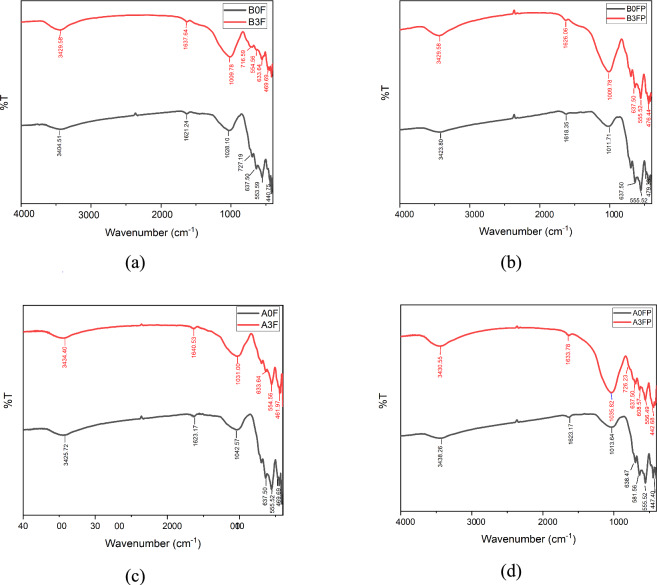
FTIR spectra of: (**a**) Fe_3_O_4_@Fly ash NaOH non-PEG 4000; (**b**) Fe_3_O_4_@Fly ash NaOH + PEG 4000; (**c**) Fe_3_O_4_@Fly ash HCl non-PEG 4000; and (**d**) Fe_3_O_4_@Fly ash HCl + PEG 4000.

When Fe₃O₄ is combined with chemically activated fly ash to form Fe₃O₄@fly ash composites, the coexistence of aluminosilicate-related bands and Fe–O vibration bands implies intimate contact between the two phases. This structural integration provides both aluminosilicate surface sites and magnetic iron oxide sites on the adsorbent surface, which is beneficial for adsorption performance while enabling magnetic separation. The FTIR spectra of Fe₃O₄@fly ash composites activated with NaOH or HCl, prepared with and without PEG 4000, are presented in Figure 5.

The FTIR spectra of Fe₃O₄@fly ash activated with NaOH and HCl, both with and without PEG 4000, show patterns similar to those of activated fly ash and raw fly ash. The absorption band near 3400 cm⁻^1^ corresponds to O–H stretching vibrations, while the band around 1620 cm⁻^1^ indicates O–H bending, confirming the presence of surface hydroxyl groups that play an important role in adsorbing metal ions. The band at approximately 1020 cm⁻^1^ is attributed to the asymmetric stretching of Si–O, and the region between 800 and 500 cm⁻^1^ is associated with the symmetric stretching of Si–O–Si, indicating the preservation of the aluminosilicate framework after core–shell formation. The main distinction among the samples appears in the fingerprint region. The band around 555 cm⁻^1^ is characteristic of Fe–O vibrations, and the band near 470 cm⁻^1^ corresponds to Fe^2^⁺–O, confirming the successful incorporation of Fe₃O₄ into the fly ash matrix. The coexistence of Si–O/Si–O–Si vibrations from fly ash and Fe–O/Fe^2^⁺–O vibrations from magnetite demonstrates that the Fe₃O₄ particles are coated or embedded on the fly ash surface, forming a core–shell structure. These spectral features suggest that the modification process does not disrupt the silicate network of fly ash but instead introduces additional Fe–O sites that enhance the adsorbent’s affinity for Cu(II). FTIR results were used only to qualitatively identify Fe–O, Si–O, and O–H functional groups. Because FTIR cannot quantify surface functional group density or determine their specific contribution to Cu(II) adsorption, the interpretation of adsorption mechanisms remains limited. Quantitative surface analysis (e.g., XPS or Boehm titration) was not performed and is acknowledged as a methodological limitation. The presence of PEG does not significantly alter the positions of the main functional groups, indicating that PEG serves mainly as a dispersion and pore-modifying agent rather than altering the chemical framework. FTIR analysis indicates the formation of Fe₃O₄@fly ash composites with surface functional groups relevant to Cu(II) adsorption and structural features consistent with a core–shell-like configuration. The observed differences in Cu(II) adsorption performance between NaOH-activated and HCl-activated samples are primarily attributed to variations in surface functional groups and surface charge distribution induced by the respective activation treatments. FTIR analysis confirmed that NaOH activation enhanced the intensity of –OH-related bands, which are known to serve as effective binding sites for divalent metal ions such as Cu(II).

In this context, FTIR spectroscopy was employed to qualitatively identify surface functional groups present on the Fe₃O₄@fly ash composites. FTIR spectroscopy was employed to qualitatively identify surface functional groups present on the Fe₃O₄@fly ash composites. While FTIR enables identification of surface functional groups, it does not provide quantitative information on functional group density. As a result, the individual contribution of specific surface groups to Cu(II) adsorption cannot be directly quantified, and adsorption mechanisms are interpreted qualitatively based on correlations between FTIR features and comparative adsorption performance.

The enhanced Cu(II) adsorption observed for NaOH-activated samples is consistent with the increased intensity of –OH-related bands, suggesting a higher availability of surface hydroxyl sites that can participate in Cu(II) binding through surface complexation. Definitive discrimination of the contribution of individual functional groups would require quantitative surface analysis techniques such as X-ray photoelectron spectroscopy (XPS) or Boehm titration, which were beyond the scope of this study. While FTIR analysis provides information on surface functional groups relevant to Cu(II) interaction, phase identification and confirmation of crystalline iron oxide formation require structural analysis. X-ray diffraction (XRD) was employed to examine the crystalline phases of the Fe₃O₄-decorated fly ash composite.

### XRD characterization

The formation of spinel-type iron oxide observed in the XRD patterns confirms that the modified Fe^3^⁺:Fe^2^⁺ precursor ratio did not hinder Fe₃O₄ formation. In heterogeneous systems involving mineral substrates such as fly ash, deviations from the ideal stoichiometric ratio are commonly reported, as surface-assisted nucleation and the presence of intrinsic iron species can influence the effective redox balance during precipitation.These considerations support the use of the applied stoichiometry for composite synthesis rather than for bulk magnetite production.

**Fig. 6:**
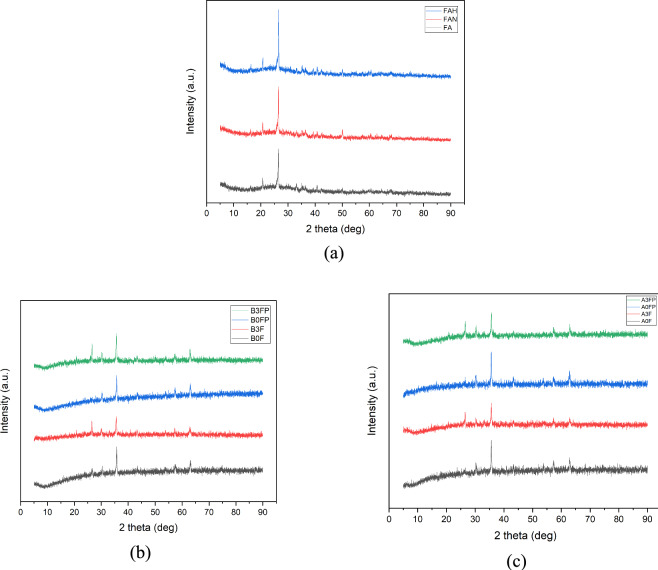
XRD characterization of: (**a**) FA, FAN, FAH; (**b**) Fe_3_O_4_@Fly ash NaOH; and (**c**) Fe_3_O_4_@Fly ash HCl.

In this context, XRD characterization was carried out to identify the crystalline phases present in raw and modified fly ash as well as in the Fe₃O₄@fly ash composites. The diffraction patterns of all samples are presented in Figure 6, where Figure 6(a) compares FA, FAN, and FAH, Figure 6(b) shows Fe₃O₄@fly ash prepared with NaOH activation, and Figure 6(c) shows Fe₃O₄@fly ash prepared with HCl activation. Because FTIR and XRD cannot reliably distinguish magnetite (Fe₃O₄) from maghemite (γ-Fe₂O₃) at the nanoscale owing to their similar spinel structures, the detected iron oxide phase is conservatively described as a spinel-type iron oxide consistent with Fe₃O₄, while acknowledging the possible presence of γ-Fe₂O₃. Although alkaline co-precipitation conditions generally favor magnetite formation, partial oxidation to γ-Fe₂O₃ during processing cannot be excluded. Definitive phase identification would require complementary techniques such as Mössbauer spectroscopy, Raman spectroscopy, or XPS, which were beyond the scope of this study.

XRD analysis in this study was employed to qualitatively identify the presence of crystalline iron oxide phases rather than to perform quantitative phase analysis. Rietveld refinement or phase percentage determination was not conducted; therefore, the relative fraction of Fe₃O₄ within the composite cannot be quantitatively determined. The diffraction results are interpreted to confirm phase presence rather than phase abundance. Quantitative phase fraction analysis would require Rietveld refinement or complementary techniques, which were beyond the scope of this study.

As seen in Figure 6 (a), the FA, FAN, and FAH samples exhibit very similar diffraction patterns, indicating that they share almost the same crystalline framework. The most intense diffraction peak appears at around 2θ ≈ 25°, which is characteristic of SiO₂ and confirms the presence of quartz as the dominant crystalline phase in the fly ash^45^. After activation with either NaOH or HCl, the intensity of this SiO₂ peak becomes more pronounced, suggesting that the activation treatments remove part of the amorphous impurities and enrich the silica-containing phase in the fly ash matrix. For the Fe₃O₄@fly ash composites shown in Figure 6(b) and 6(c), additional reflections emerge at around 2θ ≈ 35°, which are assigned to the Fe₃O₄ phase^46^. These peaks confirm that magnetite has been successfully deposited on the surface of the activated fly ash. Although XRD patterns after calcination confirmed the presence of Fe₃O₄, partial surface oxidation to γ-Fe₂O₃ at 400 °C cannot be completely excluded. Because no Rietveld refinement or complementary phase analysis was conducted, this possibility is acknowledged as a methodological limitation. The XRD results confirm the presence of Fe₃O₄ qualitatively,however, the actual Fe₃O₄ phase fraction in the composite cannot be determined because no Rietveld refinement or quantitative phase analysis was performed. This methodological limitation has been acknowledged, and the Fe₃O₄ content is therefore interpreted qualitatively. The diffraction patterns still display the broad background hump associated with the amorphous aluminosilicate glass of fly ash, indicating that the original fly ash structure is preserved and that Fe₃O₄ is present as a dispersed crystalline phase. The intensity of the Fe₃O₄ peak is higher for the samples prepared in the presence of PEG 4000, which implies improved dispersion and/or higher loading of magnetite on the fly ash surface. This combination of a silica-rich fly ash matrix and crystalline Fe₃O₄ domains supports the formation of a core–shell type composite, where the fly ash provides a porous support and surface sites for adsorption, while Fe₃O₄ imparts magnetic properties that facilitate rapid separation of the adsorbent after Cu(II) removal.

Table 2 summarizes the crystallite size of the samples estimated using the Scherrer equation. It should be emphasized that the Scherrer approach provides only an approximate crystallite size based on XRD peak broadening and cannot be used to determine crystallinity degree (%). All instances where crystallinity percentage was previously reported have been corrected, and the discussion is now restricted to crystallite size only. Variations in crystallite size among the samples suggest that chemical activation induces changes in the microstructural features of the fly ash matrix. The NaOH-treated sample (FAN) exhibits a larger apparent crystallite size compared to raw fly ash (FA), which may reflect partial restructuring of the aluminosilicate framework and altered long-range order. Crystallite size alone does not directly quantify defect density, amorphous content, or surface reactivity. Any relationship between crystallite size variation and Cu(II) adsorption behavior should be regarded as indirect and interpreted with caution, particularly in the absence of complementary textural and surface analyses.

**Table 2:** Crystallite size (nm) of the samples estimated by the scherrer equation.

**Sample**	**Crystal size (nm)**
FA	25.46
FAN	36.52
FAH	41.49
Fe_3_O_4_	22.36
B0F	29.35
B3F	43.37
B0FP	29.57
B3FP	40.76
A0F	30.82
A3F	37.70
A0FP	32.91
A3FP	32.58

The crystallite size values listed in Table 2 indicate that chemical activation produces microstructural changes in the fly ash matrix. At 400 °C, the formation of a larger number of crystal nuclei can limit final crystallite growth and lead to smaller crystallite sizes, consistent with previous reports^47^^,^^48^. The NaOH-activated sample (FAN) exhibits a larger crystallite size compared to raw fly ash (FA), suggesting partial reconstruction of the aluminosilicate framework and the development of regions with reduced long-range order. The HCl-activated sample (FAH) shows a similar trend, with crystallite size variations that reflect surface restructuring caused by acid leaching. These structural modifications are expected to influence the availability of defect sites and surface functional groups that may contribute to Cu(II) adsorption^49^.

For the composite samples, variations in crystallite size among formulations reflect differences in Fe₃O₄ loading and the role of PEG during nucleation and growth. Smaller crystallite sizes are commonly associated with enhanced nucleation during precipitation, which may lead to increased surface heterogeneity. While crystallite size alone does not quantify defect density or amorphous content, such variations may indirectly influence surface reactivity and adsorption behavior, as reported in previous studies^50^. These differences in crystallite size may help explain the observed differences in Cu(II) adsorption performance among the Fe₃O₄@fly ash composites. The role of polyethylene glycol (PEG) in the synthesis of Fe₃O₄@fly ash composites was evaluated based on comparative adsorption performance rather than direct textural measurements. Although quantitative porosity parameters such as specific surface area and pore volume were not determined, adsorption data consistently showed higher Cu(II) removal efficiencies for PEG-assisted samples compared to non-PEG counterparts prepared under identical conditions.

This improvement suggests that PEG primarily functions as a dispersion-controlling and morphology-directing agent, promoting more uniform deposition of iron oxide on the fly ash surface and reducing particle aggregation. The available data do not support a direct claim that PEG quantitatively increases porosity; therefore, its role is interpreted in terms of improved particle dispersion and surface accessibility rather than confirmed pore formation. Among the PEG-assisted samples, variations in adsorption performance were observed with different PEG molecular weights, further supporting the role of PEG in controlling particle dispersion rather than serving as a direct pore-forming agent. XRD analysis establishes the crystalline phases of the material, whereas bulk elemental composition is evaluated using XRF analysis prior to morphological examination by SEM.

### XRF characterization

X-ray fluorescence (XRF) analysis was employed to determine the bulk elemental and oxide composition of the fly ash, providing complementary compositional information to the phase identification obtained from XRD analysis. The analysis was conducted to provide compositional information on the major and minor constituents of the fly ash prior to composite preparation. Fly ash samples were collected from the Adipala coal-fired power plant during the preparation stage, and the resulting oxide and elemental compositions are summarized in Table 3.

**Table 3:** XRF-derived oxide and elemental composition of coal fly ash from the Adipala coal-fired power plant, reported to characterize bulk composition and identify the presence of major and minor elements relevant to environmental application.

**Oxide composition**	**wt%**	**Elemental composition**	**wt%**
SiO₂	13	Si	8.4
Al₂O₃	3.8	Al	2.6
Fe₂O₃	39.3	Fe	44.1
CaO	37.3	Ca	38.7
K₂O	1.1	K	1.3
TiO₂	1.1	Ti	1.0
SO₃	2.2	S	1.2
P₂O₅	0.5	P	0.3
MnO	0.49	Mn	0.59
SrO	0.661	Sr	0.941
ZrO₂	0.064	Zr	0.071
BaO	0.32	Ba	0.46

The XRF results show that the fly ash consists predominantly of SiO₂ (13 wt%), Al₂O₃ (3.8 wt%), Fe₂O₃ (39.3 wt%), and CaO (37.3 wt%), accompanied by minor oxide components including K₂O, TiO₂, SO₃, P₂O₅, MnO, SrO, ZrO₂, and BaO. Elemental composition analysis indicates that iron and calcium are the dominant elements, followed by silicon and aluminum. According to the classification criteria defined in ASTM C618, fly ash is categorized into Class C, Class F, or Class N based on the combined contents of SiO₂, Al₂O₃, and Fe₂O₃, as well as the CaO fraction. The compositional profile presented in Table 3 indicates that the Adipala fly ash is classified as Class C fly ash, characterized by a relatively high CaO content (>10 wt%) and a combined SiO₂–Al₂O₃–Fe₂O₃ content below 70 wt%. Class C fly ash is typically derived from lignite or sub-bituminous coal combustion and exhibits both pozzolanic and cementitious properties.

The presence of silica- and alumina-rich phases in the fly ash is associated with surface functional groups, including hydroxyl-containing sites, which are known to participate in adsorption processes involving metal ions^51^^,^^52^. A high CaO content favors the formation of calcium aluminosilicate glassy phases, representing reactive amorphous components in Class C fly ash that contribute to heavy metal uptake through surface interaction and precipitation-related mechanisms^53^. Calcium-containing phases may also interact with dissolved metal species in aqueous systems, leading to partial immobilization of heavy metal ions and a reduction in their aqueous concentration^54^. These compositional characteristics support the suitability of the investigated fly ash as a precursor material for adsorption-based applications.

The elevated iron content identified by XRF reflects the intrinsic mineralogical characteristics of the fly ash rather than the presence of externally introduced contaminants. High iron fractions have been reported to influence subsequent material processing steps, particularly in zeolite synthesis, where iron-rich phases may reduce ion-exchange capacity or promote the formation of non-target crystalline phases^55^. Thermal treatment or acid activation is therefore commonly applied as a pretreatment strategy to modify iron distribution and mitigate the influence of residual impurities prior to further material transformation^55^^,^^56^. In this study, chemical activation using NaOH or HCl was applied following fly ash preparation to adjust surface chemistry and reduce the influence of residual impurities.

XRF analysis provides bulk compositional information and does not directly assess the mobility or leaching behavior of trace elements under aqueous conditions. The absence of unusually high concentrations of hazardous trace elements in the bulk composition indicates a reduced likelihood of secondary pollution risks under controlled laboratory adsorption conditions. Comprehensive evaluation of long-term environmental safety, including leaching tests and detailed trace element speciation, remains beyond the scope of the present study and represents an important direction for future investigations, particularly for large-scale or field-based applications. Following bulk compositional assessment, surface morphology and microstructural features of the fly ash before and after chemical activation were examined using scanning electron microscopy (SEM).

### SEM characterization

**Fig. 7:**
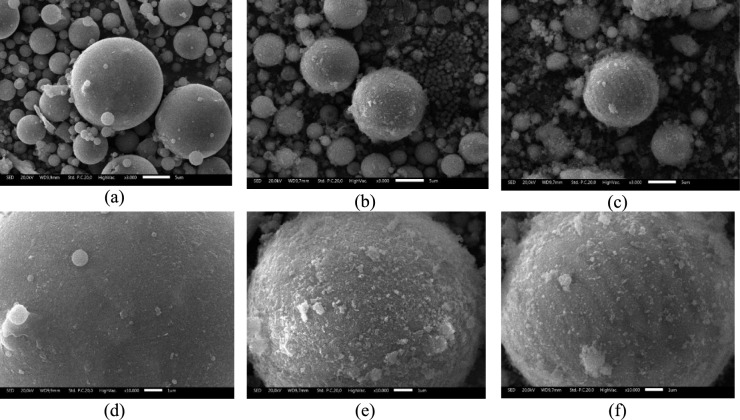
The morphology of: (**a**) FA 3000x magnification; (**b**) FAN 3000x magnification; (**c**) FAH 3000x magnification; (**d**) FA 10000x magnification; (**e**) FAN 10000x magnification; and (**f**) FAH 10000x magnification.

SEM analysis was conducted to observe changes in the surface morphology of raw fly ash and chemically activated fly ash. The SEM micrographs of FA, FAN, and FAH at magnifications of 3000× and 10 000× are presented in Figure 7.

As shown in Figure 7(a) and 7(d), the raw fly ash (FA) consists mainly of spherical particles with smooth and compact surfaces, which is typical of fly ash formed by high-temperature combustion followed by rapid cooling. This dense and glassy outer layer reflects the original aluminosilicate matrix and provides only limited surface roughness. After activation with NaOH (FAN) and HCl (FAH), clear changes in surface texture are observed (Figures 7(b), 7(c), 7(e), and 7(f)). The particles become rougher and more irregular, indicating partial dissolution and restructuring of the outer shell. This morphological change is consistent with the increased amorphous fraction inferred from the XRD results and suggests that the activation treatments disrupt the original glassy layer and expose new microstructural features. On the NaOH-activated sample, a distinct whitish layer appears on the particle surface (Figure 7(e)), which can be associated with alkali-modified silicate and aluminate phases. On the HCl-activated sample, fine lines and etching marks are visible (Figure 7(f)), indicating selective leaching of certain oxides and the development of additional surface defects. These SEM observations confirm that chemical activation transforms the initially smooth fly ash particles into surfaces with higher roughness and a greater density of potential adsorption sites. The increase in textural complexity is expected to enhance the interaction between the fly ash-based adsorbent and Cu(II) ions in solution, supporting the improved adsorption performance discussed in the subsequent sections.

SEM observations indicate that iron oxide particles are deposited on the surface of fly ash particles, resulting in a heterogeneous surface morphology. The SEM images reveal irregular particle shapes and non-uniform surface features, suggesting that the magnetic phase is distributed unevenly across the fly ash substrate. Owing to the intrinsic spatial resolution limitation of SEM, detailed verification of the nanoscale arrangement and interfacial structure between the iron oxide phase and the fly ash matrix cannot be conclusively resolved using SEM alone. TEM analysis provides direct nanoscale evidence of the distribution of iron oxide domains on the fly ash surface, confirming that the composite structure is best described as a surface-decorated magnetic composite rather than a uniform and continuous core–shell architecture. The combined SEM and TEM observations demonstrate that Fe₃O₄ is present as discrete nanoparticles and small aggregates anchored to the fly ash-derived support. Accordingly, the term “core–shell-like” is used in a descriptive sense to indicate the deposition of a magnetic phase onto the fly ash substrate, rather than to imply the formation of a perfectly concentric and homogeneous shell layer. This terminology is consistent with previous reports on magnetite-decorated mineral and fly ash-based composites where the magnetic phase is heterogeneously distributed on the support surface.

**Fig. 8:**
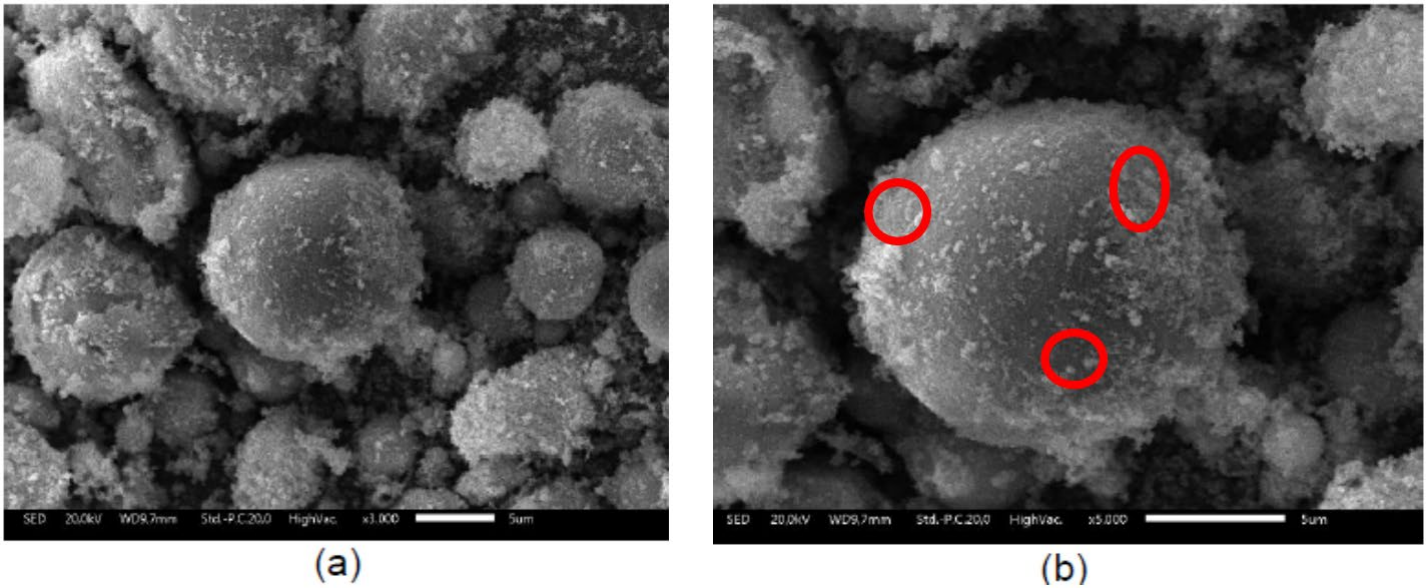
The SEM result of B0F sample: (**a**) 3000x magnification and (**b**) 5000x magnification.

To gain further insight into the morphology of the most effective adsorbent, SEM analysis was performed on the BOF sample, which showed the highest Cu(II) removal. The SEM micrographs of BOF at magnifications of 3000× and 5000× are presented in Figure 8.

The SEM images in Figure 8 show that the BOF adsorbent consists of nearly spherical particles with surfaces covered by smaller granules and deposits. Sample BOF was prepared from 0.75 g of NaOH-activated fly ash and 3 g of Fe₃O₄, and this composition yielded the highest adsorption efficiency, reaching 79.6%. The ball-like morphology, together with the increased surface roughness, indicates that Fe₃O₄ has been successfully distributed on the fly ash particle surfaces, resulting in a surface-decorated magnetic composite. Although the SEM images suggest that Fe₃O₄ is distributed on the fly ash surface in a manner consistent with core–shell-like characteristics, the intrinsic spatial resolution of SEM does not allow conclusive verification of the nanoscale interfacial architecture. At higher magnification (Figure 8(b)), several bright regions highlighted by red circles can be distinguished from the surrounding matrix. These regions are attributed to Fe₃O₄ aggregates deposited on the fly ash surface, indicating that the magnetic phase is present primarily as surface-decorated domains rather than as a uniform and continuous coating. Variations in particle size and surface texture relative to unmodified fly ash may be influenced by the relative amounts of fly ash, Fe₃O₄, and PEG 4000 used during synthesis, leading to increased surface heterogeneity that may contribute to differences in Cu(II) adsorption behavior among the samples.

Magnetic behavior of the Fe₃O₄@fly ash composites was qualitatively assessed during synthesis and post-adsorption separation by applying an external magnetic field, demonstrating that the material can be readily recovered from the aqueous phase. While quantitative magnetic parameters such as saturation magnetization were not determined, the observed magnetic response confirms the practical feasibility of magnetic separation for the composite. Transmission electron microscopy (TEM) was employed to clarify the nanoscale structural features inferred from SEM observations, particularly the distribution of the iron oxide phase on the fly ash-based matrix.

### TEM characterization

**Fig. 9:**
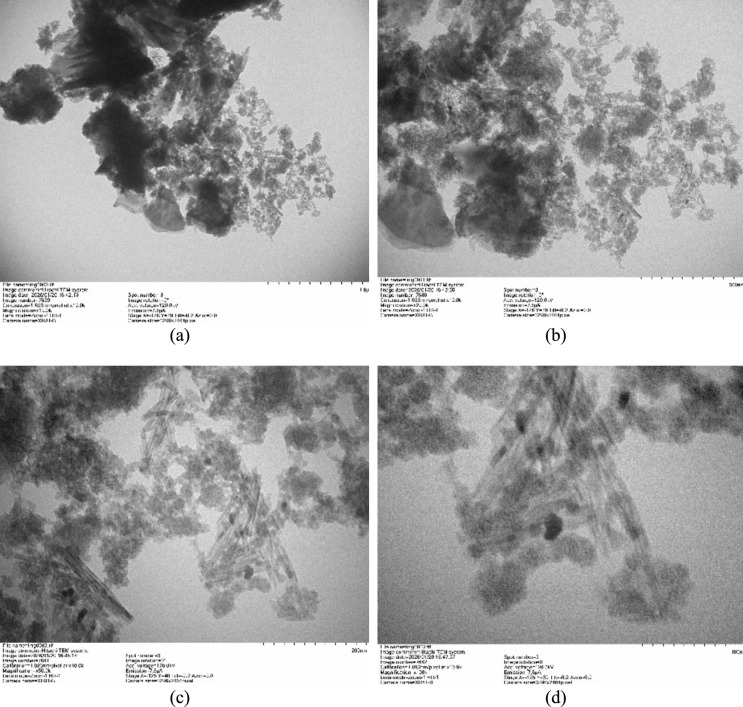
Transmission electron microscopy (TEM) images of FeOCZ acquired at different magnifications, showing the nanoscale morphology and distribution of electron-dense Fe₃O₄ domains on the fly ash-based matrix: (**a**) low-magnification overview, (**b**) intermediate magnification, (**c**) higher magnification highlighting surface-decorated regions, and (**d**) high-magnification image revealing nanoscale features of the composite. Scale bars are indicated in each panel.

Transmission electron microscopy (TEM) was employed to provide nanoscale insight into the morphology of FeOCZ and to verify the distribution of the iron oxide phase on the fly ash-derived matrix, as illustrated by the representative TEM images shown in Figure 9.

As shown in Figure 9(a), the composite exhibits irregularly shaped aggregates with regions of pronounced electron contrast, indicating the coexistence of phases with different electron densities. The darker domains are attributed to iron oxide, while the lighter regions correspond to the fly ash-derived matrix. This contrast confirms the successful incorporation of Fe₃O₄ within the composite material. At intermediate magnification (Figure 9(b)), the electron-dense regions are observed to be distributed across the support surface rather than forming a continuous layer. The heterogeneous distribution suggests that Fe₃O₄ is present as discrete nanoparticles and small aggregates attached to the fly ash surface. Such a morphology is characteristic of surface-decorated composites and differs from an idealized, uniform core–shell structure. Higher-magnification images (Figure 9(c–d)) further reveal that the iron oxide phase appears as nanoscale particles anchored to the support, with occasional agglomeration. The absence of a uniform coating thickness and the presence of exposed support regions indicate that the composite structure is dominated by localized Fe₃O₄ domains rather than a complete encapsulation of the fly ash particles. This nanoscale arrangement increases the interfacial contact between iron oxide and the surrounding matrix while preserving the heterogeneous surface features of the support. The TEM observations are consistent with the textural characteristics obtained from BET and BJH analyses, which indicated a mesoporous structure with accessible surface area. The presence of dispersed iron oxide nanoparticles on the fly ash matrix is expected to contribute additional adsorption-accessible sites and facilitate interactions between Cu(II) species and the composite surface. Following structural verification at the nanoscale by TEM, quantitative determination of Cu(II) concentrations was carried out using atomic absorption spectroscopy (AAS) to evaluate adsorption performance.

### Atomic absorption spectroscopy (AAS) analysis

AAS was employed to quantitatively determine Cu(II) concentrations in standard solutions and sample solutions, providing an analytical basis for evaluating adsorption performance. The use of multiple standard concentrations ensures reliable calibration and addresses the requirement for concentration variation in quantitative adsorption analysis.

### AAS calibration curve

**Fig. 10:**
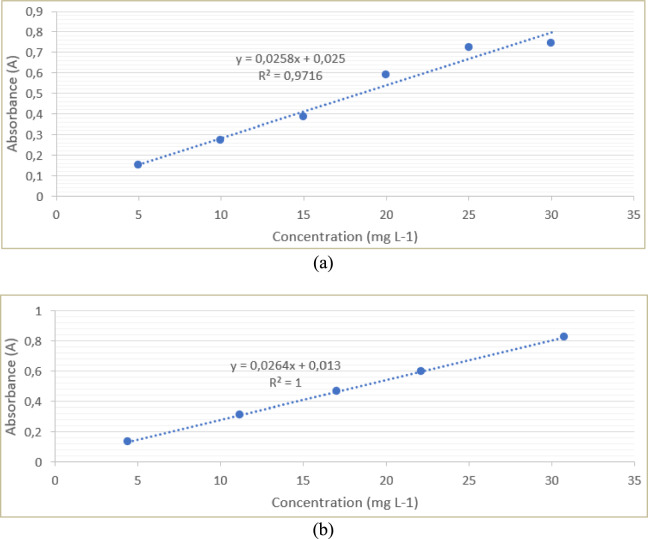
AAS calibration curves for Cu(II): (**a**) absorbance versus concentration for standard solutions and (**b**) absorbance versus calculated concentration for sample solutions.

 A series of Cu(II) standard solutions with concentrations ranging from 5 to 30 mg L⁻^1^ were prepared to construct the calibration curve. As shown in Figure 10(a), a linear relationship between absorbance and Cu(II) concentration was obtained, described by the regression equation:

$$A = 0.0258C + 0.025$$

with a coefficient of determination (R^2^ = 0.9716), indicating good linearity within the investigated concentration range. This result confirms that the AAS method is suitable for quantitative determination of Cu(II) concentrations relevant to the adsorption experiments.

In addition, a calibration plot based on the measured sample solutions is shown in Figure 10(b). The sample-based calibration also exhibits linear behavior, with a regression equation of:

$$A=0.0264C+0.013$$

and R^2^ = 1.000, further supporting the consistency of the absorbance–concentration relationship for Cu(II) within the studied range.

### AAS concentration data

Sing the calibration equation derived from the standard solutions, absorbance values measured for the sample solutions were converted to Cu(II) concentrations. The absorbance values and corresponding Cu(II) concentrations obtained for both the standard solutions and the sample solutions are presented in Table 4.

**Table 4:** AAS calibration and sample analysis data for Cu(II).

**Solution type**	**Concentration (mg L**^-1^**)**	**Absorbance**
Standard	5	0.1516
	10	0.2713
	15	0.3849
	20	0.5876
	25	0.7208
	30	0.7452
Sample	4.4067	0.1293
	11.1685	0.3076
	17.0303	0.4622
	22.1083	0.5962
	30.7692	0.8246

The standard solutions cover a concentration range of 5–30 mg L⁻^1^, while the sample solutions span from 4.41 to 30.77 mg L⁻^1^. This range demonstrates that the adsorption experiments were conducted over multiple and well-distributed concentration levels rather than at a single initial concentration. The overlap between the concentration ranges of the standards and the samples confirms that the calibration curve is directly applicable to the quantitative analysis of the adsorption data. The consistency between the absorbance–concentration relationships obtained from the standard solutions and those derived from the sample measurements supports the reliability and internal consistency of the AAS results.

### Interpretation and relevance toS adsorption analysis

The good linearity of the calibration curves and the consistency between the standard and sample absorbance data confirm the reliability of the AAS measurements. The use of multiple Cu(II) concentration levels directly addresses methodological concerns regarding insufficient concentration variation and provides a sound quantitative basis for adsorption analysis. The AAS-derived Cu(II) concentration data were subsequently used to calculate adsorption performance parameters, including removal efficiency and adsorption capacity. The availability of concentration data across a broad range enables meaningful evaluation of adsorption behavior and supports subsequent modeling using non-linear adsorption isotherms.

### Adsorption performance of Cu(II)

**Fig. 11:**
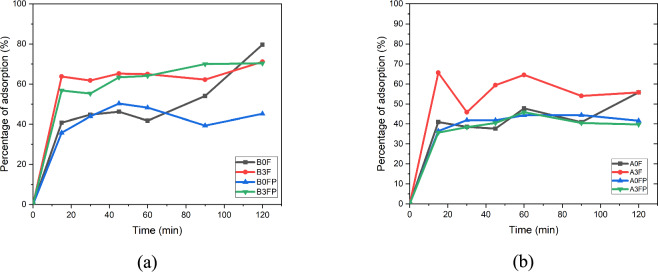
Percentage of Cu(II) removal as a function of contact time using Fe₃O₄@fly ash composites prepared from (**a**) NaOH-activated fly ash and (**b**) HCl-activated fly ash.

Adsorption performance of FeOCZ toward Cu(II) was evaluated based on removal efficiency, adsorption kinetics, and equilibrium adsorption capacity obtained under fixed experimental conditions. The assessment emphasizes comparative adsorption behavior under identical conditions rather than adsorption isotherm analysis. Batch adsorption experiments were conducted at room temperature using 0.1 g of adsorbent to evaluate adsorption performance and kinetic behavior. The effect of contact time on Cu(II) removal for Fe₃O₄@fly ash composites prepared from NaOH-activated and HCl-activated fly ash is shown in Figure 11. Figure 11(a) corresponds to Fe₃O₄@fly ash based on NaOH activation, whereas Figure 11(b) presents the adsorption behavior of composites prepared using HCl-activated fly ash.

As shown in Figure 11, Cu(II) removal increases rapidly during the initial contact period for all investigated samples, followed by a slower increase as the adsorption process approaches equilibrium. The pronounced initial uptake indicates adsorption on readily accessible active sites located on the external surface of the adsorbent particles. With increasing contact time, the adsorption rate decreases, suggesting that diffusion of Cu(II) ions into less accessible pores and interior sites becomes more influential. Among the investigated samples, the BOF composition (0.75 g NaOH-activated fly ash + 3 g Fe₃O₄) exhibits the highest Cu(II) removal efficiency, reaching a maximum removal of 79.6% at a contact time of 120 min. The superior performance of BOF is attributed to the favorable distribution of Fe₃O₄ domains on the fly ash surface and the increased availability of active adsorption sites, as supported by structural characterization.

Kinetic evaluation based on regression parameters indicates that the pseudo-second-order (PSO) model provides a better description of Cu(II) adsorption for most samples compared with the pseudo-first-order (PFO) model. This observation suggests that surface chemical interactions, such as surface complexation or ion exchange, play a significant role in the adsorption process. The comparison between kinetic models is based on fitted parameters and correlation coefficients rather than visual inspection of fitted curves. Equilibrium adsorption capacity (qₑ) was calculated at the equilibrium contact time, providing a quantitative measure of Cu(II) uptake under the investigated conditions. Variations in qₑ among the samples reflect differences in surface accessibility and active site availability. The observed adsorption performance is consistent with the physicochemical characteristics of FeOCZ. The mesoporous texture revealed by BET analysis facilitates mass transfer, while SEM and TEM observations confirm the surface-decorated distribution of Fe₃O₄ domains on the fly ash matrix. FTIR analysis further indicates the presence of surface hydroxyl and Fe–O functional groups that can participate in Cu(II) binding.

Adsorption performance in this study was evaluated under fixed experimental conditions to enable comparative assessment of removal efficiency and kinetic behavior among the investigated samples. While adsorption isotherm analysis typically requires equilibrium data collected over a range of initial concentrations, the present results provide a reliable basis for evaluating the adsorption behavior of Cu(II) on FeOCZ within the investigated experimental scope.

## Conclusion

In this study, a magnetic Fe₃O₄-decorated fly ash composite was successfully prepared and evaluated for Cu(II) removal from aqueous solutions. The adsorption behavior was investigated under controlled batch conditions with emphasis on removal efficiency and adsorption kinetics. The kinetic data were better described by the pseudo-second-order model, indicating that surface-related interactions play an important role in the Cu(II) adsorption process. Preliminary equilibrium analysis performed under fixed initial concentration conditions indicated that the Freundlich model provided a more appropriate empirical description than the Langmuir model, suggesting heterogeneous adsorption characteristics on the composite surface within the investigated experimental range. Among the investigated formulations, the BOF composite, prepared with a PEG:fly ash:Fe₃O₄ mass ratio of 0:1:4, exhibited the highest Cu(II) removal efficiency, achieving an adsorption percentage of 79.7% and an adsorption capacity of 0.318 mg g⁻^1^ at a contact time of 120 min. Although the obtained adsorption capacity is relatively modest compared with engineered high–surface-area adsorbents, the results provide insight into the influence of chemical activation and Fe₃O₄ loading on the structural characteristics and adsorption behavior of fly ash–based composites. The findings highlight both the potential and the limitations of Fe₃O₄-decorated fly ash as a low-cost, waste-derived magnetic adsorbent. The composite demonstrates practical magnetic recoverability and moderate Cu(II) removal efficiency, while constraints related to surface area, porosity development, and adsorption capacity remain evident. The magnetic separability of the composite enables efficient solid–liquid separation after adsorption and may reduce the risk of secondary particle dispersion during water treatment processes. Further performance improvement is expected to depend on enhanced textural modification, more uniform dispersion of the magnetic phase, and systematic adsorption studies over broader concentration ranges and more complex water matrices. Within this context, Fe₃O₄-decorated fly ash represents a viable platform for coal fly ash valorization and a foundation for the development of improved magnetic adsorbents for wastewater treatment applications.

## Supplementary Information

Supplementary Information 1.

Supplementary Information 2.

Supplementary Information 3.

## Data Availability

The datasets generated and analyzed during the current study are available from the corresponding author upon reasonable request.
