# Supplementary material for: Valorization of coal fly ash into a magnetic Fe₃O₄-decorated composite for Cu(II) removal from aqueous systems
Source: Sci Rep. 2026 Mar 5;16:12098. doi: 10.1038/s41598-026-41916-2 (PMC13076866; doi:10.1038/s41598-026-41916-2)
Supplement: Supplementary file 1 — Supplementary Information 1. [file 41598_2026_41916_MOESM1_ESM.pdf]

UNS Universitas Sebelas Maret Active!	**SUB LAB KIMIA**<br>**UPT LABORATORIUM PUSAT MIPA**<br>**UNIVERSITAS SEBELAS MARET**

**Nomor**	**1.308/FG-2.1/2014**	**FORM DATA PEMAKAIAN INSTRUMEN**
**Tanggal terbit**	**01 Januari 2014**	
**Revisi**	**01**	
**Halaman**	**1 dari 1**	

DATA PEMAKAIAN AAS

No. **: 067** **Tanggal : 22-01-26**

Nama : Laila Nur Fitria **NIM :K3322061**

Fak/Jur **: FKIP /Pendidikan Kimia UNS (TIDAK AKSES)**

Parameter	Jumlah			Print
	STD	Jumlah sampel	Total	
Cu	5	5	10	

Teknis/Laboran **Mahasiswa**

(……………………) (……………….)

Operator Name: WINDOWS 10
Results File: C:\Users\WINDOWS 10\Documents\Analisa cU-22-01-2026.-1.SLR

Report Date: 22/01/2026 13:43:41

General Parameters

Method : Analisis Cu
Autosampler : None
Use SFI: No

Operator : WINDOWS 10

Instrument Mode: Flame
Dilution: None

Analysis Details

Analysis Name: Analysis 1 22/01/2026
Operator Name: WINDOWS 10

Spectrometer: iCE 3000 05202804 v1,30

Solution Results - Cu

Y = 0,02637x + 0,0131
Fit: 0,9821
Characteristic Conc: 0,1668

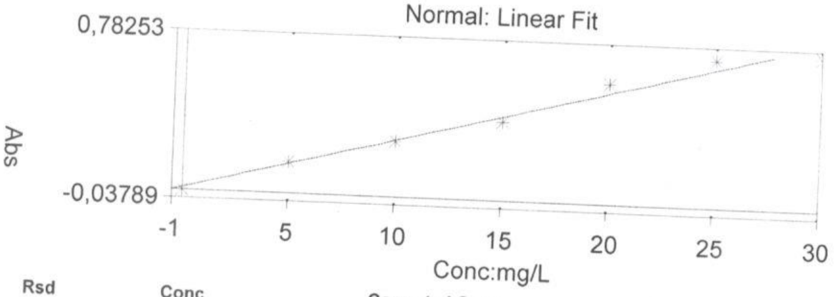

Sample ID		Signal Abs	Rsd %	Conc mg/L		Corrected Conc mg/L		Auto Dilution
Cu Blank		-0,0006	>99	0,0000				
	1	0,0002			Background: -0,0004		22/01/2026 13:37:00	
	2	-0,0005			Background: 0,0003		22/01/2026 13:37:02	
	3	-0,0014			Background: 0,0011		22/01/2026 13:37:05	
Cu Standard 1		0,1516	1,1	5,0000				
	1	0,1531			Background: 0,0017		22/01/2026 13:37:20	
	2	0,1497			Background: 0,0024		22/01/2026 13:37:22	
	3	0,1520			Background: 0,0021		22/01/2026 13:37:24	
Cu Standard 2		0,2713	4,4	10,0000				
	1	0,2642			Background: 0,0018		22/01/2026 13:37:39	
	2	0,2849			Background: 0,0032		22/01/2026 13:37:41	
	3	0,2647			Background: 0,0030		22/01/2026 13:37:44	
Cu Standard 3		0,3849	0,2	15,0000				
	1	0,3846			Background: 0,0032		22/01/2026 13:38:01	
	2	0,3858			Background: 0,0035		22/01/2026 13:38:04	
	3	0,3842			Background: 0,0039		22/01/2026 13:38:06	
Cu Standard 4		0,5876	0,1	20,0000				
	1	0,5873			Background: 0,0048		22/01/2026 13:38:24	
	2	0,5875			Background: 0,0049		22/01/2026 13:38:26	
	3	0,5880			Background: 0,0044		22/01/2026 13:38:29	
Cu Standard 5		0,7208	0,6	25,0000				
	1	0,7254			Background: 0,0054		22/01/2026 13:38:49	
	2	0,7191			Background: 0,0059		22/01/2026 13:38:52	
	3	0,7179			Background: 0,0048		22/01/2026 13:38:54	
Cu Standard 6		0,7452	1,4	30,0000				
	1	0,7576			Background: 0,0054		22/01/2026 13:39:14	
	2	0,7375			Background: 0,0051		22/01/2026 13:39:16	
	3	0,7407			Background: 0,0059		22/01/2026 13:39:18	
Cu 10 ppm		0,1293	0,9	4,4067		4,4067		1,000
	1	0,1289			Background: 0,0034		22/01/2026 13:39:59	
	2	0,1284			Background: 0,0033		22/01/2026 13:40:01	
	3	0,1307			Background: 0,0035		22/01/2026 13:40:04	
Cu 20 ppm		0,3076	3,7	11,1685		11,1685		1,000
	1	0,3144			Background: 0,0049		22/01/2026 13:40:21	
	2	0,3139			Background: 0,0053		22/01/2026 13:40:24	
	3	0,2946			Background: 0,0050		22/01/2026 13:40:26	
Cu 30 ppm		0,4622	6,3	17,0303		17,0303		1,000
	1	0,4818			Background: 0,0060		22/01/2026 13:40:42	
	2	0,4761			Background: 0,0063		22/01/2026 13:40:44	
	3	0,4287			Background: 0,0058		22/01/2026 13:40:46	
Cu 40 ppm		0,5962	5,3	22,1083		22,1083		1,000
	1	0,5861			Background: 0,0058		22/01/2026 13:41:01	
	2	0,6318			Background: 0,0061		22/01/2026 13:41:04	
	3	0,5706			Background: 0,0063		22/01/2026 13:41:06	
Cu [illegible]		0,5847	1,9	21,6757		21,6757		1,000
	1	0,5963			[illegible]			

SOLAAR AA Report

Operator Name: WINDOWS 10
Results File: C:\Users\WINDOWS 10\Documents\Analisa cU-22-01-2026.-1.SLR

Report Date: 22/01/2026 13:43:41

Solution Results - Cu

Sample ID		Signal	Rsd	Conc	Corrected Conc	Auto Dilution
		Abs	%	mg/L	mg/L	
Cu 50 ppm		0,8246	0,3	30,7692 C	30,7692 C	1,000
	1	0,8224	Background: 0,0065		22/01/2026 13:42:37	
	2	0,8275	Background: 0,0068		22/01/2026 13:42:39	
	3	0,8238	Background: 0,0067		22/01/2026 13:42:41	
