# Supplementary figures and images for: Valorization of coal fly ash into a magnetic Fe₃O₄-decorated composite for Cu(II) removal from aqueous systems
Source: Sci Rep. 2026 Mar 5;16:12098. doi: 10.1038/s41598-026-41916-2 (PMC13076866; doi:10.1038/s41598-026-41916-2)

| Larutan Standar |            | Larutan Sampel  |            |
|-----------------|------------|-----------------|------------|
| Konsentrasi (C) | Absorbansi | Konsentrasi (C) | Absorbansi |
| 5               | 0.1516     | 4.4067          | 0.1293     |
| 10              | 0.2713     | 11.1685         | 0.3076     |
| 15              | 0.3849     | 17.0303         | 0.4622     |
| 20              | 0.5876     | 22.1083         | 0.5962     |
| 25              | 0.7208     | 30.7692         | 0.8246     |
| 30              | 0.7452     |                 |            |

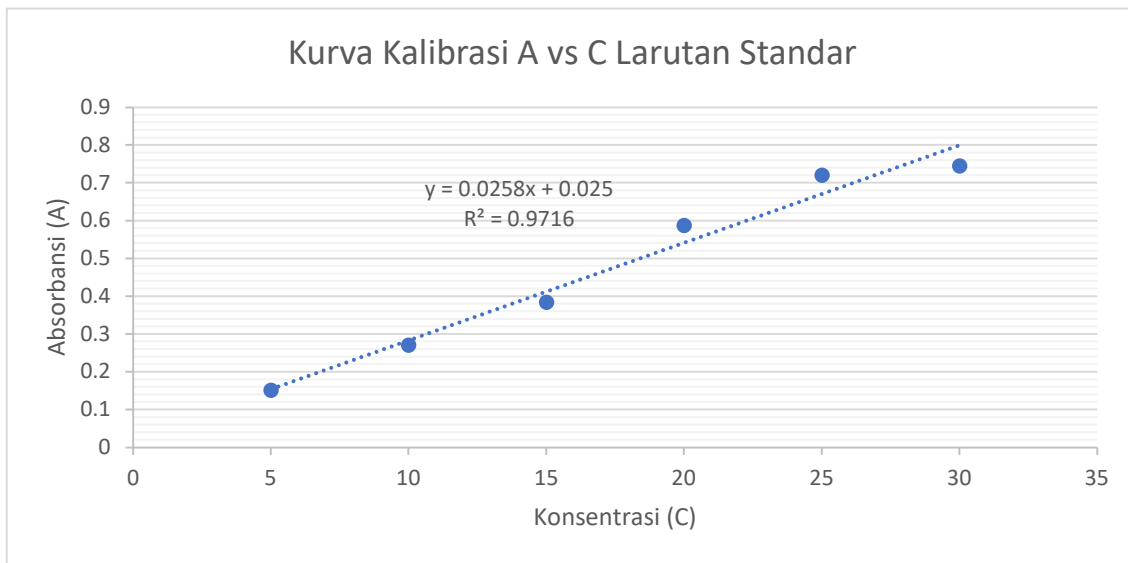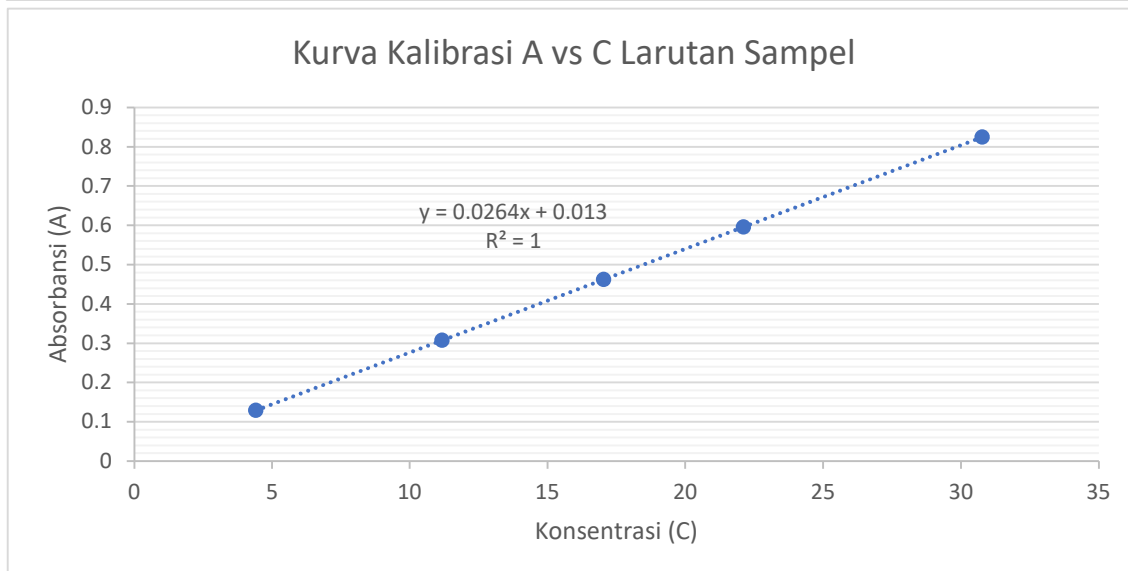

Supplement: Supplementary file 2 — Supplementary Information 2. [file 41598_2026_41916_MOESM2_ESM.pdf]
