# Supplementary material for: Valorization of coal fly ash into a magnetic Fe₃O₄-decorated composite for Cu(II) removal from aqueous systems
Source: Sci Rep. 2026 Mar 5;16:12098. doi: 10.1038/s41598-026-41916-2 (PMC13076866; doi:10.1038/s41598-026-41916-2)
Supplement: Supplementary file 3 — Supplementary Information 3. [file 41598_2026_41916_MOESM3_ESM.pdf]

Report date: 01/23/2026 **Operator:** nova
File Name: 20260115-0905_FeOCZ_4_RUN_20260120_1.qcuPhysIso

Analysis Data

Sample

**ID**	20260115-0905_FeOCZ	**Weight**	1.2981 g		
**Name**	20260115-0905_FeOCZ				

Analysis

**Data ID**	{7fec9145-e44c-4f7a-95fd-2c8c4227fc75}				
**Analysis Profile**	-N2 full Isotherm -25-25-2024 A-D				
**Operator**	nova	**Date**	01/20/2026	**Duration**	894.98 min
**Instrument**	St 4 on NOVA 800 [s/n:1050060228]			**Firmware**	1.05
**Ambient Temp.**	27.24 °C	**Void Volume Mode**	NOVA Mode	**Cell ID**	B8-4 st4
**Sample Volume**	0.000 cm³	**Sample Volume Mode**	Measured	**Cell Type**	9 mm with filler rod
**Thermal Delay**	600 sec	**p_0 Mode**	Monitor		

Adsorbate

**Name**	Nitrogen	**Molecular Weight**	28.0134 g/mol	**Cross Sectional Area**	16.2 Å²/molecule
**Non-Ideality**	6.58e-05 1/Torr	**Bath Temperature**	77.35 K		

Degas information

**Type**	Vacuum Degassing
**Operator**	
**Degassed**	at 200 °C for 6 hours

Report date: 01/23/2026 **Operator:** nova
File Name: 20260115-0905_FeOCZ_4_RUN_20260120_1.qcuPhysIso

Isotherm-Linear

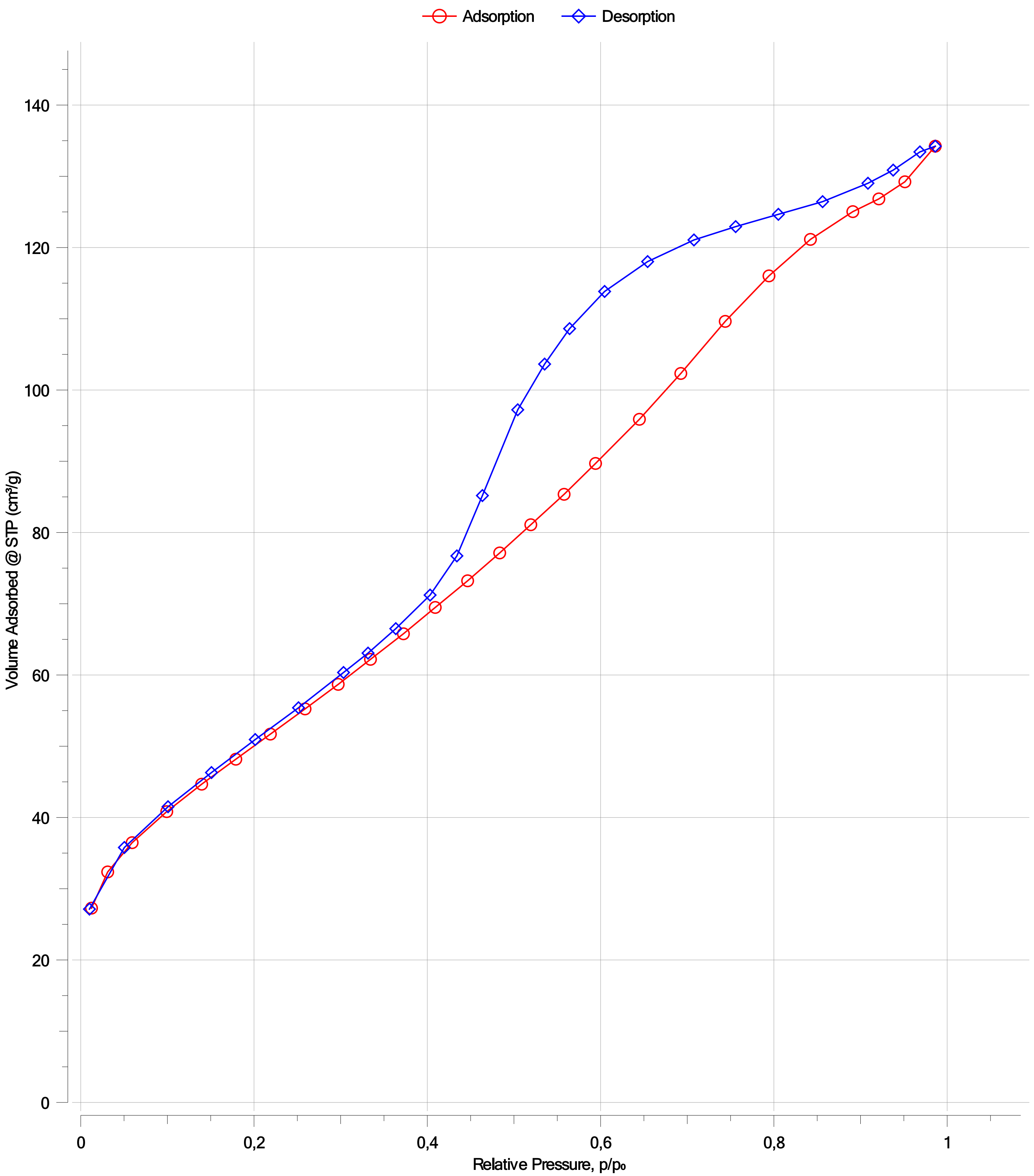

Report date: 01/23/2026 **Operator:** nova
File Name: 20260115-0905_FeOCZ_4_RUN_20260120_1.qcuPhysIso

BET-Multipoint BET

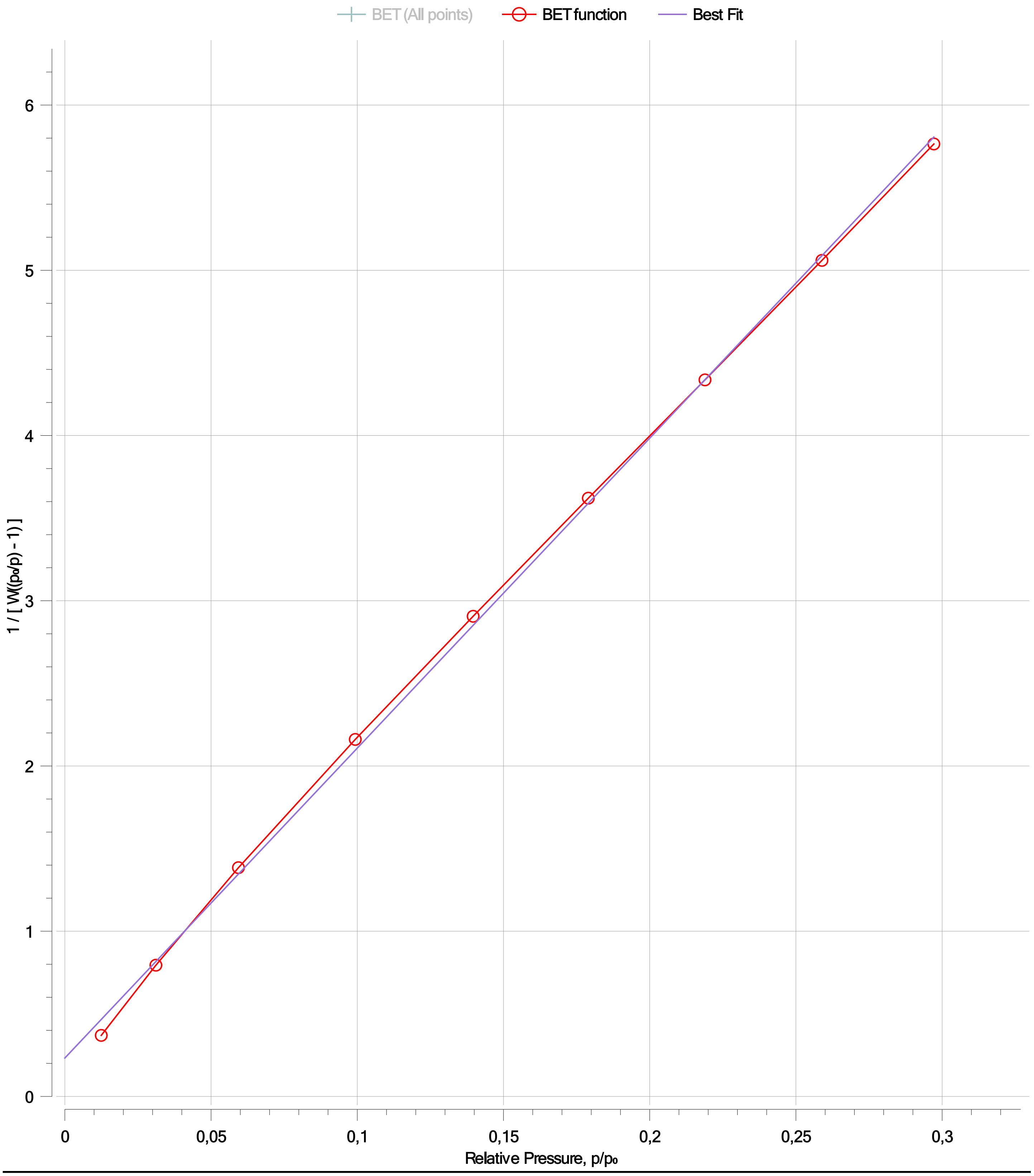

Report date: 01/23/2026 **Operator:** nova
File Name: 20260115-0905_FeOCZ_4_RUN_20260120_1.qcuPhysIso

BET-Single-point BET

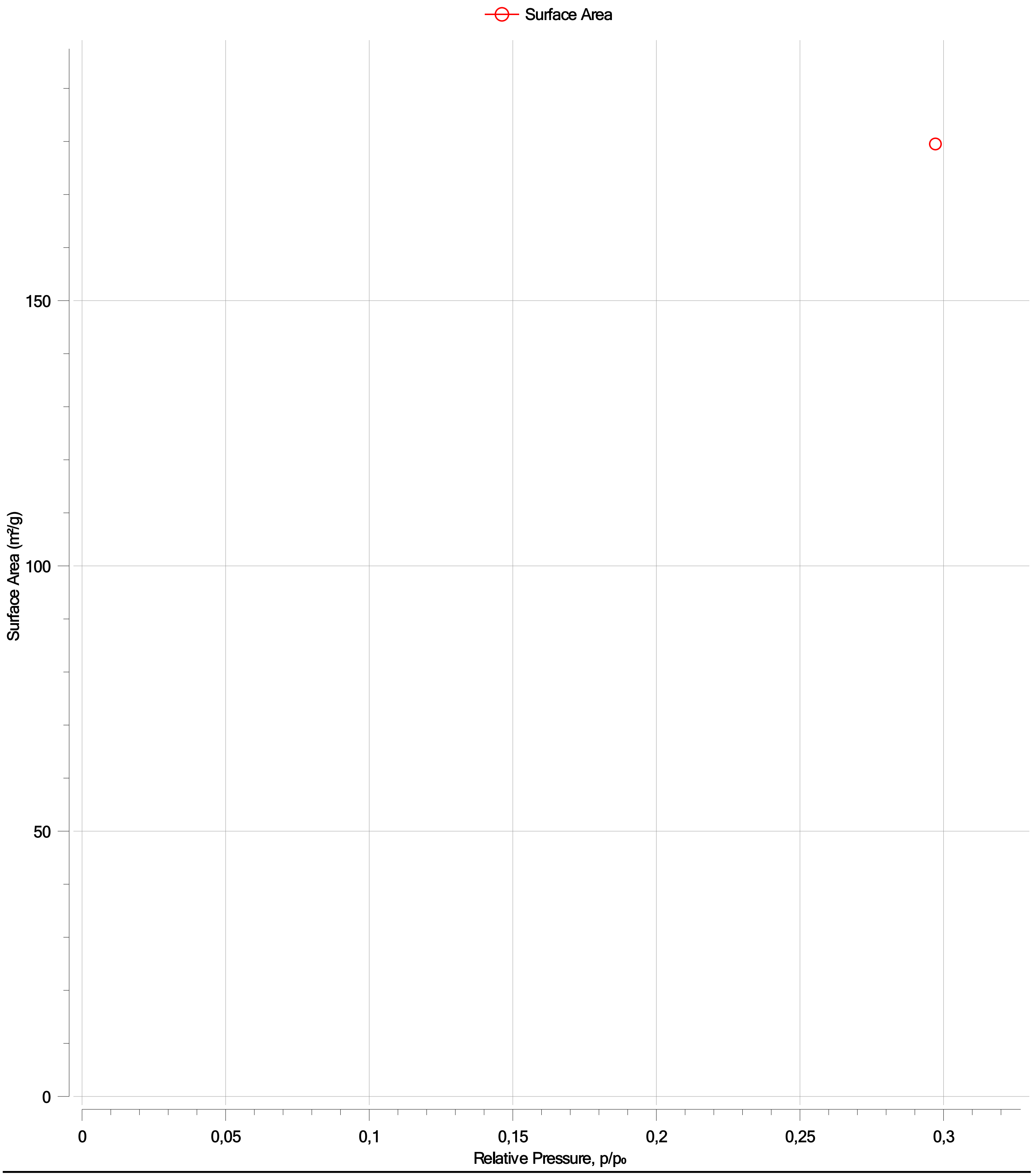

Report date: 01/23/2026 **Operator:** nova
File Name: 20260115-0905_FeOCZ_4_RUN_20260120_1.qcuPhysIso

BJH-Adsorption-dV(d)

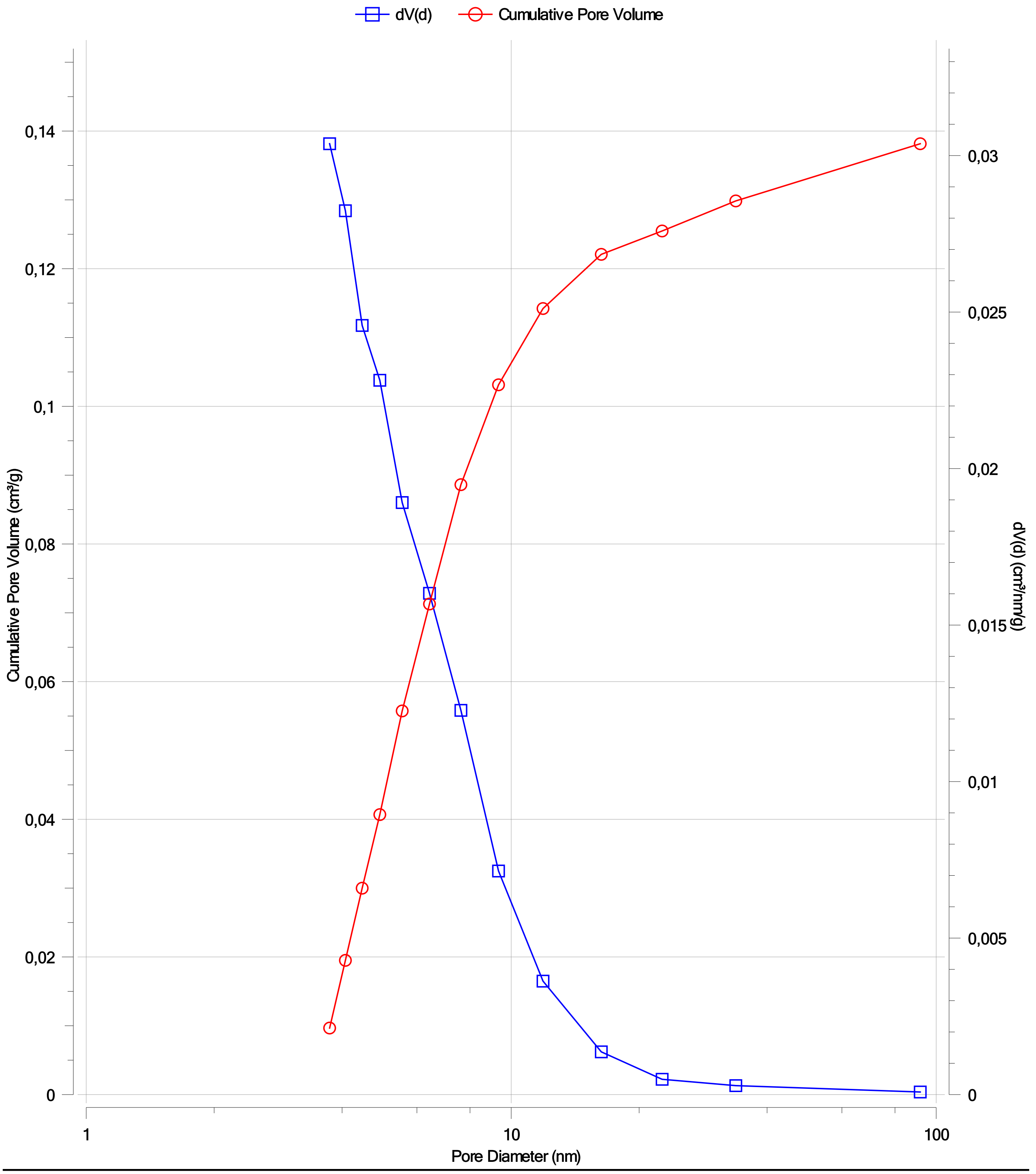

Report date: 01/23/2026 **Operator:** nova
File Name: 20260115-0905_FeOCZ_4_RUN_20260120_1.qcuPhysIso

BJH-Desorption-dV(d)

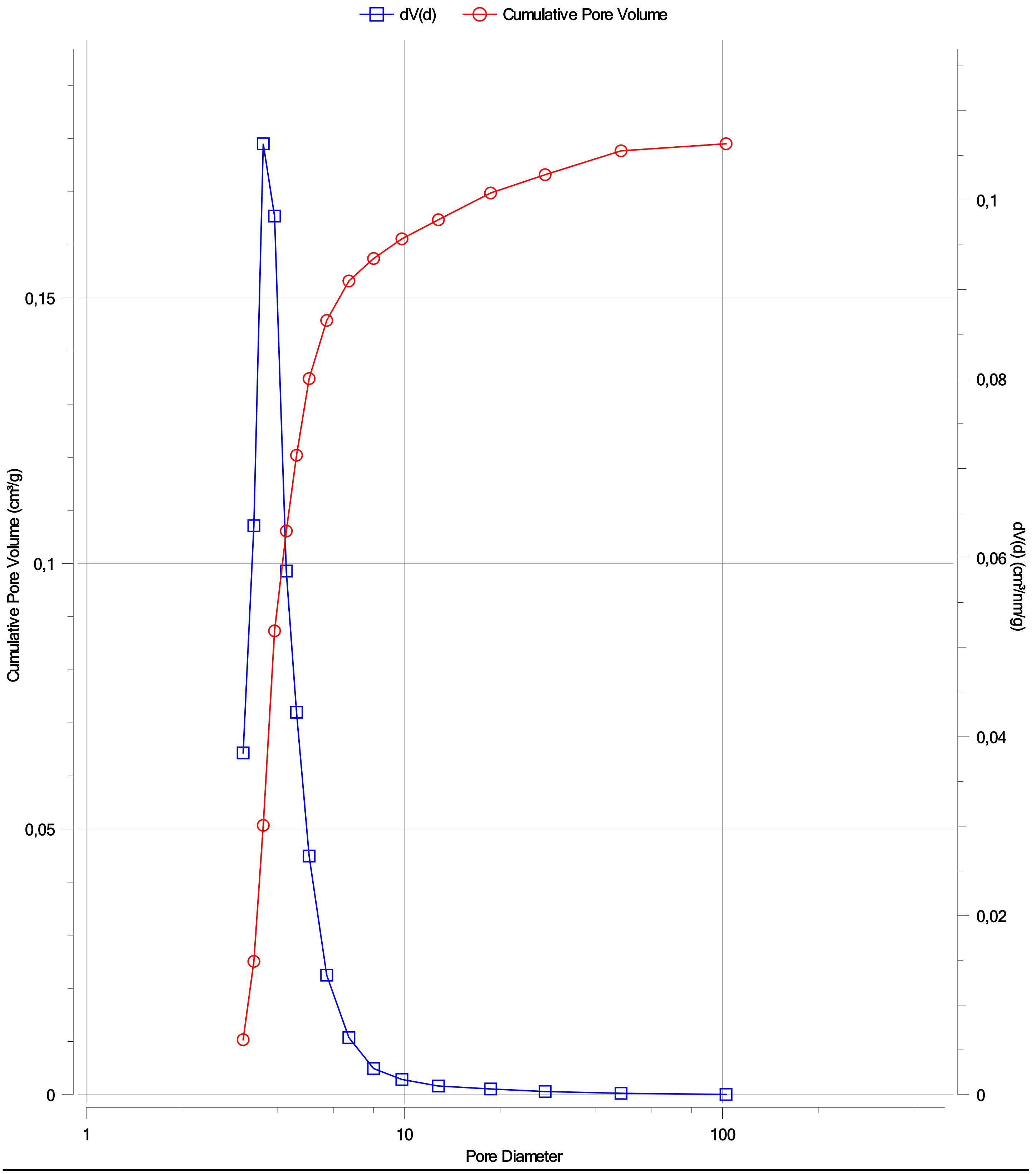

Report date: 01/23/2026 **Operator:** nova
File Name: 20260115-0905_FeOCZ_4_RUN_20260120_1.qcuPhysIso

DA Method Pore Volume

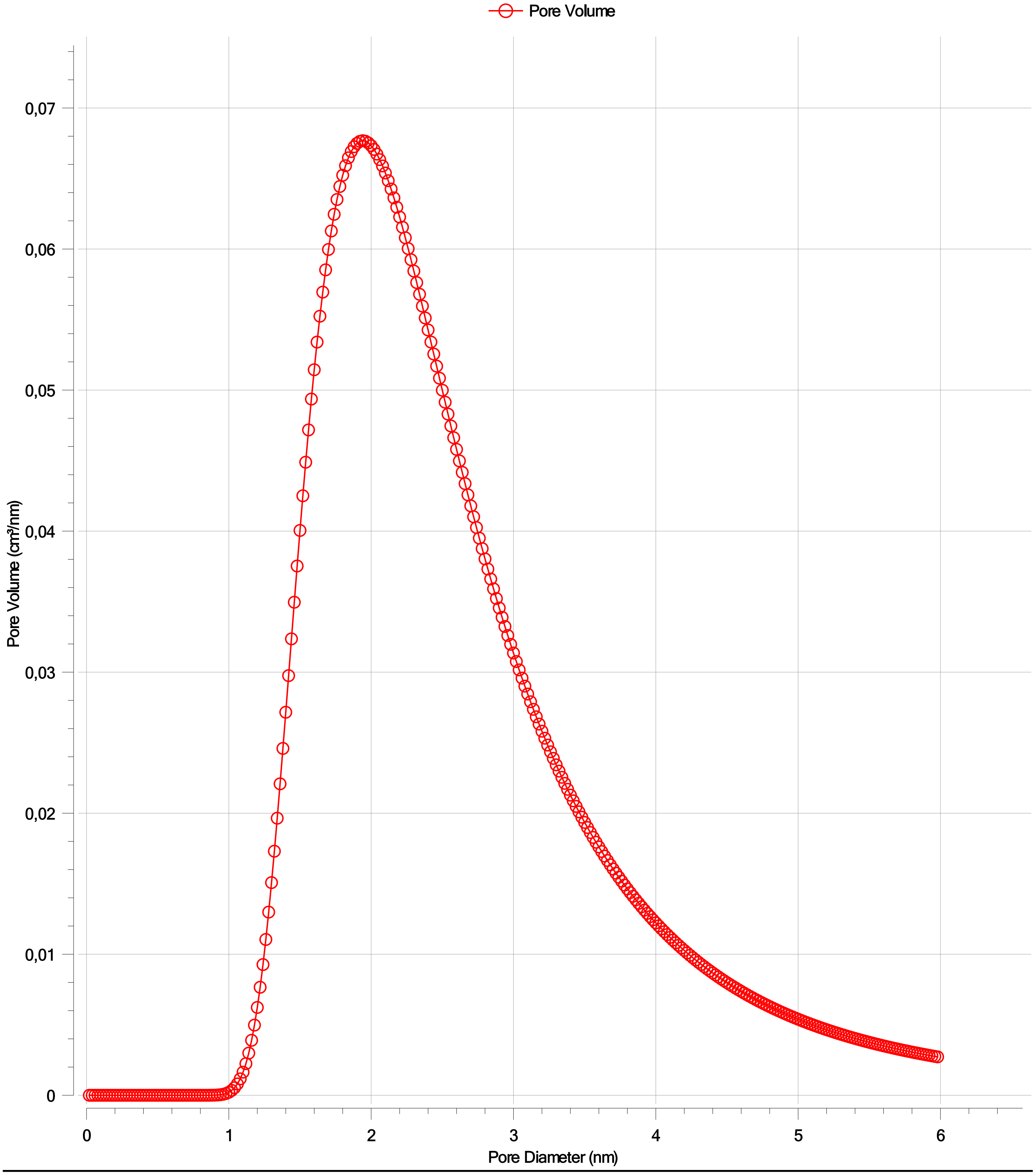

Report date: 01/23/2026 **Operator:** nova
File Name: 20260115-0905_FeOCZ_4_RUN_20260120_1.qcuPhysIso

Isotherm-Relative

Relative Pressure, p/p_0	Volume Adsorbed @STP cm^3/g
0.0124401	27.2735
0.0311272	32.3581
0.0593553	36.4654
0.0993006	40.8244
0.139608	44.6756
0.178999	48.1790
0.218894	51.7024
0.258896	55.2393
0.297162	58.6852
0.334420	62.1979
0.372440	65.7860
0.409163	69.4824
0.446547	73.2232
0.483555	77.1334
0.519451	81.0913
0.557881	85.3621
0.594174	89.6944
0.644791	95.8918
0.692576	102.325
0.743855	109.637
0.794332	116.021
0.842195	121.144
0.891115	125.040
0.921156	126.828
0.951192	129.230
0.986262	134.225
0.968530	133.422
0.937695	130.868
0.908575	129.025
0.856231	126.442
0.805188	124.664
0.755884	122.937
0.707746	121.066
0.654282	118.028
0.604634	113.836
0.564207	108.619
0.535385	103.640
0.504376	97.2261
0.463596	85.1852
0.434138	76.7164
0.403220	71.2193
0.363639	66.5063
0.331604	63.0733
0.303296	60.3512
0.251340	55.4007
0.201405	50.9400
0.150900	46.3058
0.100756	41.5075
0.0503746	35.7882
0.0100749	27.1392

BET-Single-point BET

Relative Pressure, p/p_0	Volume Adsorbed @STP cm^3/g	$1 / [W((p_0/p) - 1)]$	Slope	Surface Area m^2/g
0.297162	58.6852	5.7645	19.3984	179.526

Report date: 01/23/2026 **Operator:** nova
File Name: 20260115-0905_FeOCZ_4_RUN_20260120_1.qcuPhysIso

BET-Multipoint BET

Relative Pressure, p/p_0	Volume Adsorbed @STP cm^3/g	$1 / [W((p_0/p) - 1)]$
0.0124401	27.2735	0.3695
0.0311272	32.3581	0.7944
0.0593553	36.4654	1.3845
0.0993006	40.8244	2.1607
0.139608	44.6756	2.9060
0.178999	48.1790	3.6208
0.218894	51.7024	4.3367
0.258896	55.2393	5.0600
0.297162	58.6852	5.7645

BJH Pore Size Distribution-Desorption

Diameter nm	Pore Volume cm^3/g	Pore Surface Area m^2/g	dV(d) $cm^3/nm/g$	dS(d) $m^2/nm/g$	dV(log d) cm^3/g	dS(log d) m^2/g
3.11384	1.033833e-02	1.328049e+01	3.819813e-02	4.906888e+01	2.737034e-01	3.515962e+02
3.36509	2.508282e-02	3.080690e+01	6.359313e-02	7.559157e+01	4.925505e-01	5.854826e+02
3.60152	5.070014e-02	5.925860e+01	1.062970e-01	1.180580e+02	8.811710e-01	9.786662e+02
3.90848	8.732696e-02	9.674303e+01	9.821259e-02	1.005122e+02	8.832047e-01	9.038847e+02
4.25543	1.061116e-01	1.144001e+02	5.852510e-02	5.501210e+01	5.731862e-01	5.387805e+02
4.58293	1.203893e-01	1.268617e+02	4.274393e-02	3.730706e+01	4.508595e-01	3.935118e+02
5.02053	1.348197e-01	1.383588e+02	2.666568e-02	2.124532e+01	3.079616e-01	2.453620e+02
5.70110	1.457718e-01	1.460430e+02	1.335644e-02	9.371133e+00	1.750309e-01	1.228050e+02
6.69449	1.532049e-01	1.504843e+02	6.370511e-03	3.806420e+00	9.795002e-02	5.852574e+01
8.00523	1.574390e-01	1.526000e+02	2.910722e-03	1.454411e+00	5.350456e-02	2.673481e+01
9.82593	1.611381e-01	1.541059e+02	1.691585e-03	6.886203e-01	3.811370e-02	1.551555e+01
12.7970	1.647400e-01	1.552317e+02	9.591306e-04	2.997986e-01	2.805792e-02	8.770155e+00
18.6742	1.697734e-01	1.563099e+02	6.292461e-04	1.347838e-01	2.663808e-02	5.705844e+00
27.7093	1.732171e-01	1.568070e+02	3.419392e-04	4.936085e-02	2.157446e-02	3.114396e+00
47.9970	1.777129e-01	1.571817e+02	1.473835e-04	1.228272e-02	1.572455e-02	1.310460e+00
102.646	1.790440e-01	1.572336e+02	1.689376e-05	6.583282e-04	3.788446e-03	1.476309e-01

BJH Pore Size Distribution-Adsorption

Diameter nm	Pore Volume cm^3/g	Pore Surface Area m^2/g	dV(d) $cm^3/nm/g$	dS(d) $m^2/nm/g$	dV(log d) cm^3/g	dS(log d) m^2/g
3.73911	9.669888e-03	1.034458e+01	3.037799e-02	3.249753e+01	2.613852e-01	2.796226e+02
4.07238	1.950215e-02	2.000209e+01	2.823675e-02	2.773491e+01	2.646145e-01	2.599116e+02
4.45991	2.999102e-02	2.940934e+01	2.457251e-02	2.203858e+01	2.521502e-01	2.261483e+02
4.90756	4.068139e-02	3.812273e+01	2.282076e-02	1.860050e+01	2.576804e-01	2.100273e+02
5.53995	5.574351e-02	4.899799e+01	1.891418e-02	1.365656e+01	2.408573e-01	1.739056e+02
6.42332	7.128375e-02	5.867539e+01	1.601451e-02	9.972738e+00	2.364073e-01	1.472182e+02
7.61469	8.862113e-02	6.778272e+01	1.227546e-02	6.448302e+00	2.146129e-01	1.127362e+02
9.33658	1.031312e-01	7.399917e+01	7.142815e-03	3.060140e+00	1.529505e-01	6.552740e+01
11.8809	1.142131e-01	7.773015e+01	3.624822e-03	1.220386e+00	9.861391e-02	3.320081e+01
16.2923	1.220841e-01	7.966260e+01	1.365161e-03	3.351668e-01	5.067429e-02	1.244127e+01
22.6522	1.254812e-01	8.026247e+01	4.885011e-04	8.626110e-02	2.527816e-02	4.463700e+00
33.7650	1.298490e-01	8.077990e+01	2.860070e-04	3.388202e-02	2.185178e-02	2.588687e+00
91.7223	1.381584e-01	8.114228e+01	8.256352e-05	3.600588e-03	1.551966e-02	6.768112e-01

DA Method Micropore Analysis

Diameter nm	dV(d) $cm^3/nm/g$
0.020000	4.11138e-28
0.040000	2.56961e-29
0.060000	5.07578e-30
0.080000	1.60601e-30
0.100000	6.57821e-31
0.120000	3.17236e-31

continues on next page

Report date: 01/23/2026 **Operator:** nova
File Name: 20260115-0905_FeOCZ_4_RUN_20260120_1.qcuPhysIso

DA Method Micropore Analysis continued...

Diameter nm	dV(d) cm³/nm/g
0.140000	1.71236e-31
0.160000	1.00376e-31
0.180000	6.26640e-32
0.200000	4.11138e-32
0.220000	2.80813e-32
0.240000	1.98273e-32
0.260000	1.43951e-32
0.280000	1.07023e-32
0.300000	8.12125e-33
0.320000	6.27347e-33
0.340000	4.92257e-33
0.360000	3.91650e-33
0.380000	3.15481e-33
0.400000	2.56961e-33
0.420000	2.11403e-33
0.440000	1.75508e-33
0.460000	1.46919e-33
0.480000	1.23920e-33
0.500000	7.72765e-33
0.520000	3.80598e-29
0.540000	5.48088e-26
0.560000	2.85370e-23
0.580000	6.36680e-21
0.600000	6.98053e-19
0.620000	4.20395e-17
0.640000	1.52329e-15
0.660000	3.57960e-14
0.680000	5.80495e-13
0.700000	6.84163e-12
0.720000	6.11998e-11
0.740000	4.30950e-10
0.760000	2.46379e-09
0.780000	1.17402e-08
0.800000	4.76842e-08
0.820000	1.68278e-07
0.840000	5.24574e-07
0.860000	1.46520e-06
0.880000	3.71246e-06
0.900000	8.62495e-06
0.920000	1.85457e-05
0.940000	3.72112e-05
0.960000	7.01718e-05
0.980000	0.000125155
1.00000	0.0002123
1.02000	0.000344189
1.04000	0.000535655
1.06000	0.000803328
1.08000	0.00116497
1.10000	0.00163866
1.12000	0.00224185
1.14000	0.00299043
1.16000	0.0038979
1.18000	0.00497461
1.20000	0.00622723
1.22000	0.00765842
1.24000	0.00926669
1.26000	0.0110465
1.28000	0.0129884
1.30000	0.0150797
1.32000	0.0173047
1.34000	0.0196453

continues on next page

Report date: 01/23/2026 **Operator:** nova
File Name: 20260115-0905_FeOCZ_4_RUN_20260120_1.qcuPhysIso

DA Method Micropore Analysis continued...

Diameter nm	dV(d) cm³/nm/g
1.36000	0.0220817
1.38000	0.0245929
1.40000	0.0271575
1.42000	0.0297538
1.44000	0.0323607
1.46000	0.0349578
1.48000	0.0375259
1.50000	0.040047
1.52000	0.0425051
1.54000	0.0448855
1.56000	0.0471755
1.58000	0.0493642
1.60000	0.0514423
1.62000	0.0534024
1.64000	0.0552387
1.66000	0.0569469
1.68000	0.0585244
1.70000	0.0599694
1.72000	0.0612817
1.74000	0.0624621
1.76000	0.0635121
1.78000	0.0644343
1.80000	0.0652317
1.82000	0.0659082
1.84000	0.0664678
1.86000	0.0669151
1.88000	0.0672551
1.90000	0.0674926
1.92000	0.0676331
1.94000	0.0676817
1.96000	0.0676438
1.98000	0.0675247
2.00000	0.0673297
2.02000	0.067064
2.04000	0.0667325
2.06000	0.0663402
2.08000	0.0658919
2.10000	0.0653922
2.12000	0.0648454
2.14000	0.0642558
2.16000	0.0636275
2.18000	0.0629641
2.20000	0.0622695
2.22000	0.0615469
2.24000	0.0607996
2.26000	0.0600308
2.28000	0.0592431
2.30000	0.0584394
2.32000	0.0576221
2.34000	0.0567936
2.36000	0.0559559
2.38000	0.0551112
2.40000	0.0542614
2.42000	0.053408
2.44000	0.0525528
2.46000	0.0516973
2.48000	0.0508426
2.50000	0.0499902
2.52000	0.049141
2.54000	0.0482962
2.56000	0.0474567

continues on next page

Report date: 01/23/2026 **Operator:** nova
File Name: 20260115-0905_FeOCZ_4_RUN_20260120_1.qcuPhysIso

DA Method Micropore Analysis continued...

Diameter nm	dV(d) cm³/nm/g
2.58000	0.0466234
2.60000	0.0457969
2.62000	0.0449779
2.64000	0.0441672
2.66000	0.0433652
2.68000	0.0425725
2.70000	0.0417893
2.72000	0.0410163
2.74000	0.0402535
2.76000	0.0395014
2.78000	0.0387602
2.80000	0.038030
2.82000	0.037311
2.84000	0.0366034
2.86000	0.0359073
2.88000	0.0352226
2.90000	0.0345495
2.92000	0.033888
2.94000	0.0332381
2.96000	0.0325997
2.98000	0.0319728
3.00000	0.0313574
3.02000	0.0307534
3.04000	0.0301607
3.06000	0.0295792
3.08000	0.0290088
3.10000	0.0284493
3.12000	0.0279008
3.14000	0.027363
3.16000	0.0268358
3.18000	0.0263191
3.20000	0.0258127
3.22000	0.0253165
3.24000	0.0248302
3.26000	0.0243539
3.28000	0.0238872
3.30000	0.0234301
3.32000	0.0229823
3.34000	0.0225438
3.36000	0.0221143
3.38000	0.0216937
3.40000	0.0212818
3.42000	0.0208785
3.44000	0.0204835
3.46000	0.0200968
3.48000	0.0197182
3.50000	0.0193475
3.52000	0.0189846
3.54000	0.0186292
3.56000	0.0182813
3.58000	0.0179407
3.60000	0.0176073
3.62000	0.0172808
3.64000	0.0169612
3.66000	0.0166482
3.68000	0.0163419
3.70000	0.0160419
3.72000	0.0157483
3.74000	0.0154608
3.76000	0.0151793
3.78000	0.0149037

continues on next page

Report date: 01/23/2026 **Operator:** nova
File Name: 20260115-0905_FeOCZ_4_RUN_20260120_1.qcuPhysIso

DA Method Micropore Analysis continued...

Diameter nm	dV(d) cm³/nm/g
3.80000	0.0146338
3.82000	0.0143696
3.84000	0.0141109
3.86000	0.0138575
3.88000	0.0136095
3.90000	0.0133665
3.92000	0.0131287
3.94000	0.0128957
3.96000	0.0126676
3.98000	0.0124441
4.00000	0.0122253
4.02000	0.012011
4.04000	0.011801
4.06000	0.0115954
4.08000	0.011394
4.10000	0.0111966
4.12000	0.0110034
4.14000	0.010814
4.16000	0.0106285
4.18000	0.0104467
4.20000	0.0102686
4.22000	0.0100941
4.24000	0.00992311
4.26000	0.00975554
4.28000	0.00959134
4.30000	0.00943041
4.32000	0.00927269
4.34000	0.00911811
4.36000	0.00896659
4.38000	0.00881807
4.40000	0.00867248
4.42000	0.00852976
4.44000	0.00838984
4.46000	0.00825266
4.48000	0.00811816
4.50000	0.00798629
4.52000	0.00785698
4.54000	0.00773017
4.56000	0.00760582
4.58000	0.00748387
4.60000	0.00736426
4.62000	0.00724695
4.64000	0.00713188
4.66000	0.00701901
4.68000	0.00690828
4.70000	0.00679966
4.72000	0.0066931
4.74000	0.00658854
4.76000	0.00648595
4.78000	0.00638529
4.80000	0.00628652
4.82000	0.00618959
4.84000	0.00609446
4.86000	0.0060011
4.88000	0.00590947
4.90000	0.00581953
4.92000	0.00573124
4.94000	0.00564458
4.96000	0.0055595
4.98000	0.00547598
5.00000	0.00539397

continues on next page

Report date: 01/23/2026 **Operator:** nova
File Name: 20260115-0905_FeOCZ_4_RUN_20260120_1.qcuPhysIso

DA Method Micropore Analysis continued...

Diameter nm	dV(d) cm³/nm/g
5.02000	0.00531346
5.04000	0.0052344
5.06000	0.00515677
5.08000	0.00508053
5.10000	0.00500567
5.12000	0.00493214
5.14000	0.00485993
5.16000	0.004789
5.18000	0.00471933
5.20000	0.00465089
5.22000	0.00458366
5.24000	0.00451761
5.26000	0.00445272
5.28000	0.00438896
5.30000	0.00432632
5.32000	0.00426477
5.34000	0.00420428
5.36000	0.00414484
5.38000	0.00408642
5.40000	0.00402901
5.42000	0.00397258
5.44000	0.00391712
5.46000	0.0038626
5.48000	0.00380901
5.50000	0.00375632
5.52000	0.00370453
5.54000	0.00365361
5.56000	0.00360354
5.58000	0.00355432
5.60000	0.00350591
5.62000	0.00345831
5.64000	0.00341151
5.66000	0.00336547
5.68000	0.0033202
5.70000	0.00327567
5.72000	0.00323188
5.74000	0.0031888
5.76000	0.00314642
5.78000	0.00310474
5.80000	0.00306373
5.82000	0.00302339
5.84000	0.00298369
5.86000	0.00294464
5.88000	0.00290621
5.90000	0.0028684
5.92000	0.00283119
5.94000	0.00279457
5.96000	0.00275854
5.98000	0.00272307

Report date: 01/23/2026 **Operator:** nova
File Name: 20260115-0905_FeOCZ_4_RUN_20260120_1.qcuPhysIso

Multipoint BET Summary/Results

**Isotherm Branch**	Adsorption	**Slope**	18.7564	**Intercept**	0.232552
**Correlation coeff., r**	0.999621	**C constant**	81.6544	**Surface area**	183.397 m²/g

Langmuir Method Summary/Results

**Isotherm Branch**	Adsorption	**Slope**	12.8927	**Intercept**	0.482089
**Correlation coefficient, r**	0.989682	**Surface area**	270.114 m²/g		

DR Method Summary/Results

**Slope**	-0.10039	**Intercept**	-1.13385	**Correlation coefficient, r**	0.925414
**Average Pore Width**	2.78492 nm	**Adsorption energy**	9.33598 kJ/mol	**Micropore volume**	0.0911615 cm³
**Micropore surface area**	255.882 m²/g				

MP Method Summary/Results

**BET Surface Area**	183.397 m²/g	**V-t Surface Area**	146.468 m²/g	**Total Pore Volume**	0.208 cm³/g
**MP Pore Volume**	0.150 cm³/g				

t-plot Method Summary/Results

**Isotherm Branch**	Adsorption	**Slope**	136.832 cm³/nm/g	**Intercept**	-9.59313 cm³/g
**Correlation coefficient, r**	0.999826	**Thickness method**	deBoer	**Micropore volume**	0 cm³/g
**Micropore area**	0.000 m²/g	**External surface area**	183.397 m²/g		

BJH Adsorption Summary/Results

**Surface Area**	81.1423 m²/g	**Pore Volume**	0.138158 cm³/g	**Pore Diameter**	3.73911 nm

BJH Desorption Summary/Results

**Surface Area**	157.234 m²/g	**Pore Volume**	0.179044 cm³/g	**Pore Diameter**	3.60152 nm

Kr(87K) Pore Size Distribution Summary/Results

**Pore Diameter Dv(d)**	7.23232 nm

DH Adsorption Summary/Results

**Surface Area**	82.736 m²/g	**Pore Volume**	0.134909 cm³/g	**Pore Diameter**	3.73911 nm

DH Desorption Summary/Results

**Surface Area**	160.18 m²/g	**Pore Volume**	0.174728 cm³/g	**Pore Diameter**	3.60152 nm

SF Method Summary/Results

**Mode(Diameter)**	0.452267 nm	**Micropore Volume**	0.118492 cm³/g

HK Method Summary/Results

**Mode(Pore Width)**	0.4315 nm	**Micropore Volume**	0.118091 cm³/g

DA Method Summary/Results

**Best E**	2.429 kJ/mol	**Best n**	1.000	**DA Micropore Volume**	0.125 cm³/g
**Pore Diameter (mode)**	1.940 nm				

DFT Method Summary/Results

**Pore Volume**	0.1989 cm³/g	**Surface Area**	148.3621 m²/g	**Lower Confidence Limit**	1.5640 nm
**Mode (Pore Width)**	5.0860 nm	**Fitting Error**	0.4188 %		
**Notes**	Desorption data				

FHH Method Adsorption Summary/Results

Slope -0.57677 **Intercept** 1.60834 **Correlation coefficient, r** 0.999431

Fractal Dimension with Adsorbate Surface Tension Effects

Neglected 1.26969 **Accounted for** 2.42323

FHH Method Desorption Summary/Results

Slope -0.587152 **Intercept** 1.61481 **Correlation coefficient, r** 0.99962

Fractal Dimension with Adsorbate Surface Tension Effects

Neglected 1.23854 **Accounted for** 2.41285

NK Method Adsorption Summary/Results

Slope -0.902908 **Intercept** 2.88178 **Correlation coefficient, r** 0.996396

Fractal Dimension, D 2.90291

NK Method Desorption Summary/Results

Slope -0.727275 **Intercept** 2.84712 **Correlation coefficient, r** 0.999165

Fractal Dimension, D 2.72728

Report date: 01/23/2026 **Operator:** nova
File Name: 20260115-0905_FeOCZ_4_RUN_20260120_1.qcuPhysIso

Total Pore Volume Summary/Results

Total Pore Volume 0.2081 cm^3/g **for pores smaller than** 142.04 nm (Diameter) **at relative pressure** 0.98626

Average Pore Size Summary/Results

Average Pore Diameter 4.5396 nm

Average Particle Size Summary/Results

BET Surface area 183.3971 m^2 **Average Particle Diameter** 14.8709 nm
